# Genome-wide identification and analysis of the YABBY gene family in Moso Bamboo (*Phyllostachys edulis* (Carrière) J. Houz)

**DOI:** 10.7717/peerj.11780

**Published:** 2021-07-22

**Authors:** Ruifang Ma, Bin Huang, Zhinuo Huang, Zhijun Zhang

**Affiliations:** 1 State Key Laboratory of Subtropical Forest Cultivation, Zhejiang A&F University, Hangzhou, Lin’an, China; 2 School of Forestry and Biotechnology, ZhejiangA&F University, Zhejiang, Lin’an, China

**Keywords:** Genome-wide analysis, Expression analysis, Phyllostachys edulis, Gene family, Phylogenetic analysis

## Abstract

**Background:**

The YABBY gene family is a family of small zinc finger transcription factors associated with plant morphogenesis, growth, and development. In particular, it is closely related to the development of polarity in the lateral organs of plants. Despite being studied extensively in many plant species, there is little information on genome-wide characterization of this gene family in Moso bamboo.

**Methods:**

In the present study, we identified 16 * PeYABBY* genes, which were unequally distributed on 11 chromosomes, through genome-wide analysis of high-quality genome sequences of M oso bamboo by bioinformatics tools and biotechnological tools. Gene expression under hormone stress conditions was verified by quantitative real-time PCR (qRT-PCR) experiments.

**Results:**

Based on peptide sequences and similarity of exon-intron structures, we classified the * PeYABBY* genes into four subfamilies. Analysis of putative * cis*-acting elements in promoters of these genes revealed that * PeYABBYs* contained a large number of hormone-responsive and stress-responsive elements. Expression analysis showed that they were expressed at a high level in Moso bamboo panicles, rhizomes, and leaves. Expression patterns of putative * PeYABBY* genes in different organs and hormone-treated were analyzed using RNA-seq data, results showed that some * PeYABBY* genes were responsive to gibberellin (GA) and abscisic acid (ABA), indicating that they may play an important role in plant hormone responses. Gene Ontology (GO) analyses of YABBY proteins indicated that they may be involved in many developmental processes, particularly high level of enrichment seen in plant leaf development. In summary, our results provide a comprehensive genome-wide study of the YABBY gene family in bamboos, which could be useful for further detailed studies of the function and evolution of the YABBY genes, and to provide a fundamental basis for the study of YABBY in Gramineae for resistance to stress and hormonal stress.

## Introduction

YABBY genes are zinc finger transcription factors found throughout the plant kingdom, which include a C2–C2 zinc finger structure at the N terminus and a YABBY structure (helix-loop-helix) at the C terminus (Bowman & Eshed, 2000; Kanaya, Nakajima & Okada, 2002), which is similar to the first two helix sequences of high-mobility group box (HMG-box), a specific juxtaposition of structural domains not found in other eukaryotes (Siegfried et al., 1999; Golz et al., 2004; Sieber et al., 2004; Boter et al., 2015). These two structural domains have been shown to be implicated in the specific binding of DNA (Golz et al., 2004). The YABBY protein physically interacts with STYLOSA (STY) in *Antirrhinum majus* and with LEUNIG (LUG) and LEUNIG_HOMOLOG (LUH) in *Arabidopsis* (Navarro et al., 2004; Stahle et al., 2009). The YABBY protein of *Arabidopsis* also physically interacts with SEUSS (SEU), which is a modulator of synergy with LUG (Stahle et al., 2009). These findings suggest that YABBY proteins are involved in the synthesis of the deterrent complex along with LUG/STY and SEU. According to their biochemical properties, LUG/LUH, STY or SEU mutations enhance the phenotypes caused by functional loss mutations of the YABBY genes in *A. majus* and *Arabidopsis* (Navarro et al., 2004; Stahle et al., 2009).

A study of the YABBY gene family *Arabidopsis thaliana* revealed that several members play key roles in the establishment of dorsoventral polarity, as well as lateral organ growth, development, and morphogenesis. Additionally, this family has also been implicated in both biotic and abiotic stress response (Yamada et al., 2011; Eckardt, 2010). During the establishment of the dorsoventral axis of plant lateral organs, this family of genes mainly determines the fate of distal terminal cells and influences the development of plant lateral organs (Finet et al., 2016; Bowman, 2000). Furthermore, the YABBY genes are associated with the laminar growth, as well as suppression of shoot apical meristem (SAM) pattern genes (Sarojam et al., 2010). YABBY is a type of transcription factors that is specifically found in seed plants (Kayani et al., 2019). The YABBY gene family is relatively small and has independent origins in the seed plant genealogy (Floyd & Bowman, 2007; Liu et al., 2007). According to phylogenetic analysis, five members of YABBY gene family represented by *INO* (INNER NO OUTER), *CRC* (CRABS CLAW), *YABBY2*, *YABBY3*/*FIL* (FILAMENTOUS FLOWER), and *YABBY5*, have been found in angiosperms (Lee et al., 2005; Toriba et al., 2007; Yamada, Ito & Kato, 2004; Liu et al., 2007). In dicotyledons, the *Arabidopsis* YABBY family of genes contains six closely related transcripts: *CRC*, *FIL*, *YAB2* (*YABBY2*), *YAB3* (*YABBY3*), *INO*/*YABBY4*, and *YAB5* (*YABBY5*) (Bowman, 2000; Nahar et al., 2012). Four of these (*FIL*, *YAB2*, *YAB3*, and *YAB5*) have high expression in vegetative tissues, while *FIL*, *YAB2*, and *YAB3* genes are specifically expressed at the apical end of above-ground tissues.YAB3 plays a key role in regulating abaxial conformation, growth of lateral organs and inflorescence phyllotaxy (Hou, Lin & Hou, 2020). *FIL* is involved in flower and leaf development and is functionally redundant with the *YAB3* gene (Stahle et al., 2009). In contrast, *CRC* and *INO* are expressed only in floral organs, with the former involved in the development of nectaries and the apical end of the carpel, and the latter regulating the development of the external end of the ovule (Eckardt, 2010; Ha, Jun & Fletcher, 2010; Zhang et al., 2013; Shamimuzzaman & Vodkin, 2013).

Thus far, eight YABBY family genes have been found in rice (Toriba et al., 2007; Yamaguchi et al., 2004; Navarro et al., 2004). Sequence analysis revealed the rice *DL* (DROOPING LEAF) gene is a homolog of the *Arabidopsis CRC*, which regulates the formation of flowers and leaf veins by promoting cell proliferation in the central region of rice leaves (Yamaguchi et al., 2004; Toriba et al., 2007). *OsDL* genes are not expressed polarized in the carpels and leaf primordia, whereas *CRC* is expressed in the distal end of the nectaries and carpels. *CRC* genes are mainly involved in regulating the development of *Arabidopsis* carpels and nectaries (Bowman & Smyth, 1999).

*OsYABBY5* (TONGARI-BOUSHI1, TOB1) regulates lateral organogenesis and differentiation of meristematic tissues in rice spikelets (Tanaka et al., 2012). Overexpression of *OsYABBY1* has been shown to result in a semi-dwarf phenotype that can be recovered by the exogenous application of gibberellin (GA) (Dai et al., 2007b) . Rice *OsYAB3* is involved in leaf development but does not affect polarity establishment, and the leaves of plants expressing RNAi targeting this gene exhibit twisted, multinodular, and missing ligule auricles (Lin et al., 2012). *OsYAB4* is mainly expressed in the vascular tissues of rice and may be involved in the regulation of vascular tissue development and leaf polarity establishment (Dai et al., 2007a; Liu et al., 2007).

To date, the function of the YABBY gene family has been studied in a variety of plants, for example, gene members of the YABBY family were identified in tomato (*Solanum lycopersicum* L.) (Huang et al., 2013; Han et al., 2015); YABBY family genes also have been identified in grapes (*Vitis pseudoreticulata*) (Xiang et al., 2013; Zhang et al., 2019), pak-choi (*Brassica rapa* ssp. chinensis) (Hou et al., 2019), *Bienertia sinuspersici* (Soundararajan et al., 2019), cotton (*Gossypium arboreum*) (Yang et al., 2018), maize (*Zea mays* L.) (Juarez, Twigg & Timmermans, 2004; Strable & Vollbrecht, 2019), berberidaceae (*Epimedium sagittatum*) (Sun et al., 2013), lily (*Lilium longiflorum*) (Wang et al., 2009), Chinese cabbage (*Brassica campestris* L. ssp. *pekinensis* (Lour.) Olsson) (Zhang et al., 2013), *Pisum sativum* (Fourquin et al., 2014), rapeseed (*Brassica campestris* L. ssp *chinensis var. Parachinensis*) (Zhang & Zhang, 2014), spearmint (*Mentha spicata*) (Wang et al., 2016) and wheat (*Triticum aestivum* L.) (Zhao et al., 2006). There is increasing evidence that YABBY has similar functions in nutrient storage organs in both dicots and monocots (Golz et al., 2004; Juarez, Twigg & Timmermans, 2004). For example, petunia *PhCRC1/2* expression is similar to *Arabidopsis CRC* expression in developing nectaries and carpels (Li et al., 2018; Morel et al., 2018; Lee et al., 2005).

Moso bamboo (*Phyllostachys edulis*) belongs to the genus *Phyllostachys* of Bambusoideae in Gramineae (Zhang et al., 2018), and is the most versatile and largest economic bamboo species (Ramakrishnan et al., 2020). Because of its rapid growth and development and high economic production value, it is the most widely distributed and cultivated bamboo species in China. Due to its multiple uses of bamboo shoots and timber, it has high comprehensive incomes (Peng et al., 2013a). At the same time, moso bamboo forests also have ecological values of water conservation, fertilization, soil improvement, air purification and climate regulation. Moso bamboo is an excellent model plant due to its rapid growth rate during culm development (Tao et al., 2020; Zhao et al., 2018a). A better understanding of Moso bamboo’s genome and transcriptome has the potential to yield new genetic resources for the improvement of other crop species (Peng et al., 2013b; Pan et al., 2017; Liu et al., 2019).

Despite the sequencing of the Moso bamboo genome several years ago, few YABBY genes have been reported in this species (Zhao et al., 2018b). The recognition and functional characterization of the YABBY family in Moso bamboo will help to elucidate the regulatory mechanisms. Here, we identified 16 *PeYABBY* genes that contained the YABBY conserved domain and investigated their amplification patterns, tertiary structures, protein interactions and GO enrichment analysis. Additionally, we analyzed the expression characteristics of the *PeYABBY* genes in different tissues and in response to hormonal stress. These results may provide a basis for studying the tissue specificity of *PeYABBY* genes.

## Materials & Methods

### Identification of YABBY gene family members in *P. edulis*

The complete *P. edulis* genome files was downloaded from GigaDB (Wang et al. 2021; Zhao et al., 2018b) (http://gigadb.org/dataset/100498) while additional sequences of YABBY were obtained from the Pseudomolecule Download Site webpage in the RGAP 7 database (Rice Genome Annotation Project, http://rice.plantbiology.msu.edu/) and The Arabidopsis Information Resource, version 10 (TAIR 10) (https://www.arabidopsis.org/). BLAST analysis of YABBY proteins using Hidden Markov Models (HMMs) against the protein database. In combination with the pfam database (PF04690), rigorous criterion (E-values had a cutoff of 10-5) was used to ensure the reliability of the amino acid sequences. In addition to conserved sequences, the demonstration of conserved YABBY domains was the deterministic criterion for inclusion of candidate genes into the YABBY family (Punta et al., 2012). If there were several alternative splice variants for a candidate gene, the longest variant was used to represent the candidate genes. By sorting the results and removing redundancy, the complete nucleotide and common conserved domain sequences of 16 YABBY family members were identified in the Moso bamboo database. The online analysis tools ProtParam, ProtScale, and SignalP 4.1 Server (http://www.cbs.dtu.dk/services/SignalP/) were used to determine the physical and chemical properties of each protein, such as amino acid sequence length, molecular weight (MW), and isoelectric point (pI), as well as its signal peptides.

### Phylogenetic analysis

*Arabidopsis* YABBY protein sequences were downloaded from TAIR 10, while the RGAP 7 database was used to obtain rice YABBY protein sequences. The YABBY protein sequences of *A. thaliana*, *Oryza sativa*, and *P. edulis* were used to construct a phylogenetic tree using the neighbor- joining method after multiple sequence alignment by ClustalW (Khachane, Timmis & dosSantos, 2005). For statistical reliability, the branch lengths were calculated using pair-wise estimates of the genetic distances in a bootstrap analysis of 1,000 replicates.

### Gene structure, motifs and conserved domains-sequences analysis

We analyzed the structure of the YABBY family with the gene structure view based on the GFF annotation file. To better characterize the secondary structure of the YABBY proteins, the software MEME (Multiple Expectation Maximization for Motif Elicitation, https://meme-suite.org/meme/) was used to identify conserved motifs (Nakano et al., 2006). The following optimization parameters were used: any number of repeats, the maximum number of patterns was set to 5, and the length of each search was from 6 to 50 amino acid. The icons for the motifs were created using an online website (http://weblogo.berkeley.edu/logo.cgi).

For conserved domain-sequence analysis, the corresponding sequences of YABBY proteins were extracted and analyzed by multi-sequence alignment using Geneious software version 11.1.4 (Liao et al., 2015), and combined with an online website for conserved domains (such as NCBI Conserved Domain Database (CDD), https://www.ncbi.nlm.nih.gov/cdd/ and EMBL-Pfam, https://pfam.xfam.org/) to analyze conserved domains of the Moso bamboo YABBY sequence.

### Determination of chromosomal localization and synteny analysis

To determine the physical locations of *PeYABBY* genes, all genes of bamboo on each chromosome were obtained from the *P. edulis* genomic database, followed by mapping of the physical location information obtained from the database by using TBtools.

Collinearity analysis was performed using the Basic Local Alignment Search Tool (BLAST) (Lugli et al., 2020) to compare the entire Moso bamboo YABBY protein sequence, with a cutoff of truncated *E*-value of 1 ×10^−20^. This truncated *E*-value was also used in the collinearity analysis of *P. edulis* and rice, as well as *P. edulis* and *A. thaliana*. BLASTP results were analyzed using MCScanX software to generate collinearity blocks for the entire genome (Wang et al., 2012). CIRCOS software was used to extract colinear gene pairs from the YABBY protein family and plot the resulting collinearity (Krzywinski et al., 2009). To estimate divergence times, synonymous (Ks) and nonsynonymous (Ka) substitution rates and their corresponding cDNA sequences for the *PeYABBY* gene pairs in Moso bamboo species were calculated using the Ka/Ks feature of the TBtools software (Chen et al., 2020). The divergence time of the gene pairs was estimated using the synonymous mutation rate of *λ* substitutions per year per synonymous site, with the following formula: T = Ks/2 *λ* (*λ* = 6.5 ×10-9) (Peng et al., 2013a)

### *Cis* -acting elements in the promoter regions of the *PeYABBY* genes

For *cis*-acting regulatory elements analysis in the promoter regions of the YABBY genes, the upstream sequence (1.5 Kb) of each *PeYABBY* transcription initiation sequence was downloaded from the genome database. These sequences were then submitted to the PlantCARE database (http://bioinformatics.psb.ugent.be/webtools/plantcare/html/) for identifying *cis*-acting elements and functional categorization, followed by visualization in TBtools (Lescot et al., 2002; Chen et al., 2020).

### Expression patterns of transcriptome analysis

Transcriptomics data were obtained from the NCBI SRA database (Accession: ERX082501, ERX082502, ERX082503, ERX082504, ERX082506, ERX082507, ERX082508 and ERX082509, registration numbers ERR105067, ERR105068, ERR105069, ERR105070, ERR105072, ERR105073, ERR105074, and ERR105075) and utilized to calculate the expression level of the YABBY genes at different developmental stages, both roots, panicles, rhizomes, and leaves were also included. TopHat2 (Kim et al., 2013) was used to map paired reads to the respective reference genomes. After mapping, gene transcripts were assembled by Cufflinks (Ghosh & Chan, 2016; Kulahoglu & Brautigam, 2014). Gene expression profiles were calculated as transcripts per million reads (TPM) (Lama et al., 2020) (Table S3). For convenience, transcript expression of each gene was based on logarithm base 2 per million transcripts (log_2_ TPM), and the Amazing Heatmap module in TBtools was used to draw the gene expression heatmap (Chen et al., 2020).

### Plant growth conditions and hormone treatments

Seeds of Moso bamboo were collected from Kunming, Yunnan Province, China. The seedlings were cultivated in an experimental greenhouse with a constant photoperiod (16 h of light/8 h of darkness) and an average temperature of 22 °C for 30 days. And then four tissue samples were obtained, which consisted of roots, stems, young leaves and mature leaves, with three replicates of each sample. Abscisic acid (ABA) hormone treatment was conducted on young leaves by spraying them with a 100 µM ABA solution. After spraying, the leaves were sampled at 0 h, 3 h, 6 h, 12 h, 24 h, and 48 h. Unprocessed samples were taken at 0 h and used as the control group (CK). GA hormone treatment was conducted on young leaves by spraying with a 50 µM GA solution. Leaves were then sampled and operated like ABA treatment. All samples were immediately frozen in liquid nitrogen and then stored at −80 °C prior to RNA isolation.

### qRT-PCR analysis of *PeYABBY* genes

For quantitative real-time PCR (qRT-PCR), RNA was extracted from Moso bamboo samples with a FastPure Plant Total RNA Isolation Kit (Vazyme company, China). First-strand cDNA was generated via a HiScript@ lll 1st Strand cDNA Synthesis Kit (+gDNA wiper), following the manufacturer’s instructions. Gene-specific qRT-PCR primers were designed according to the coding sequence (CDS) of each gene by Primer Premier 5 (See Table S4 for primer sequences). qRT-PCR was performed on a CFX96 real-time system (BioRad, USA) for gene expression analysis with Hieff^®^ qPCR SYBR Green Master Mix (Yeasen company, China) buffer, and three independent replicate experiments were performed (Dong et al., 2017). PCR conditions were as follows: 94 °C for 30 s; followed by 39 amplification cycles of 94 °C for 5 s, 60 °C for 30 s, and 72 °C for 10 s.

The relative expression level of each gene was calculated and normalized through the 2^−ΔΔCt^ method relative to NTB, which was an internal control for normalization. The statistical analysis data were analyzed using SPSS software (SPSS Software, Chicago, IL, USA). The data was subjected to a one-way analysis of variance (ANOVA), and then a Tukey test was performed. Under appropriate circumstances, the non-parametric Student’s *t*-test was employed for pairwise comparisons of data from individual samples. Values of *P* ≤ 0.05 were considered significantly different.

### Protein interaction network prediction and GO enrichment

Protein-protein interaction (PPI) networks are important for studying protein interactions and functions. To further analyze the YABBY protein interactions and their interaction networks, the online software STRING (https://string-db.org/) was used to construct the relationship networks and the results were visualized using Cytoscape (Paul et al., 2003). Additionally, to better understand the biological pathways that the 16 *PeYABBY* genes were involved in, Gene Ontology (GO) enrichment analysis and visualization were performed using Gene Ontology Enrichment Analysis software (Thomson et al., 2019).

### Homology modeling

Homology modeling techniques are widely used in protein models. To determine the tertiary structure of YABBY protein, we used the fully automated protein structure homology modeling server Phyre2 database (http://www.sbg.bio.ic.ac.uk/phyre2) (Hüttner et al., 2019) for homology modeling, using similar protein structures as homology templates and visualizing them with Discovery Studio, version 2016 (BIOVIA) software.

## Results

### Identification of *PeYABBY* genes in *P. edulis*

After two rounds of genome retrieval of *P. edulis*, resulting in the identification of 16 YABBY family proteins through HMMER and the Pfam database (Accession no. PF04690), and the conservative YABBY domain was analyzed by NCBI CDD. Based on their genome location of *P. edulis*, each *PeYABBY* gene was assigned a unique name (*PeYABBY01*-*PeYABBY16*) (Table 1). The amino acid residue sequence, pI, MW and GRAVY of YABBY proteins were listed in Table 1. The predicted length of protein products varied, *PeYABBY02* had the smallest protein sequence, with 85 amino acids (aa), while *PeYABBY04* had the largest sequence (297 aa). The MW of proteins were predicted, and there were significant variations in MWs, *PeYABBY02* had a MW of 9.46 KDa and *PeYABBY04* was 31.06 KDa, and the pIs ranged from 5.55 and 9.85. Analysis of the grand average of hydropathicity (GRAVY) results indicated that the majority of the YABBY members revealed hydrophilicity, with the index <0. Only one protein (*PeYABBY02*) showing hydrophobic properties (GRAVY >0). All proteins discussed in this section were predicted to not contain signal peptides. The predicted analysis of the secondary structure of the protein sequences revealed that the largest proportion of each protein sequence was random coils, followed by alpha helices (Table S1).

**Table 1:** Physicochemical properties of *PeYABBY* genes in *P. edulis*.

**Id**	**New name**	**Number of amino acids (aa)**	**Molecular weight (KDa)**	**Theoretical pI**	**Aliphatic index**	**Grand average of hydropathicity (GRAVY)**	**Signal peptide**
PH02Gene42615.t2	*PeYABBY01*	260	28.16	9.32	82.27	−0.23	no
PH02Gene08310.t1	*PeYABBY03*	170	18.78	9.10	57.94	−0.59	no
PH02Gene41238.t1	*PeYABBY02*	85	9.46	9.55	104.24	0.63	no
PH02Gene30423.t1	*PeYABBY04*	297	31.06	9.03	75.76	−0.23	no
PH02Gene17872.t1	*PeYABBY05*	101	10.61	5.55	80.99	−0.13	no
PH02Gene42853.t1	*PeYABBY06*	122	13.85	9.85	54.34	−0.87	no
PH02Gene18063.t1	*PeYABBY07*	193	21.55	9.32	68.24	−0.55	no
PH02Gene21516.t2	*PeYABBY08*	197	21.70	8.78	68.38	−0.49	no
PH02Gene35716.t2	*PeYABBY09*	291	30.78	9.58	80.93	−0.18	no
PH02Gene11723.t1	*PeYABBY11*	193	21.66	8.71	56.06	−0.66	no
PH02Gene30480.t1	*PeYABBY10*	188	21.22	8.84	72.13	−0.41	no
PH02Gene29867.t1	*PeYABBY13*	191	21.36	8.98	57.64	−0.61	no
PH02Gene30849.t1	*PeYABBY12*	188	21.21	8.61	71.6	−0.50	no
PH02Gene09731.t1	*PeYABBY14*	252	26.86	7.06	71.47	−0.20	no
PH02Gene02055.t1	*PeYABBY16*	258	27.31	7.62	73.99	−0.17	no
PH02Gene16897.t1	*PeYABBY15*	255	27.07	6.78	74.43	−0.19	no

### Phylogenetic analysis and classification

**Figure 1:**
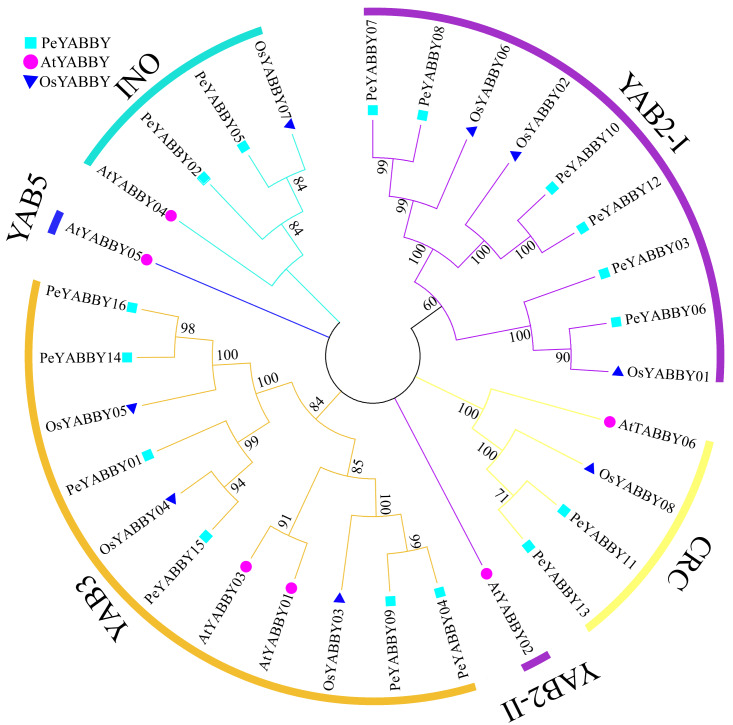
Phylogenetic tree of interspecific YABBY relationships. In MEGA 7.0, phylogenetic trees without roots were developed using the neighbor-joining (NJ) process. At each node, bootstrap values of 1,000 replicates are indicated. The phylogenetic tree resulted in a tree that was divided into five classes, with *AtYABBYs* representing the YABBY sequences of *Arabidopsis*, *OsYABBYs* representing the YABBY sequences of rice, and * PeYABBYs* representing the YABBY sequences of bamboo.

In order to gain a better understanding of the evolutionary relationships among the YABBY proteins, the full-length protein sequences of *A. thaliana*, *O. sativa* and *P. edulis* were used for multi-sequence alignment and rootless phylogenetic tree analysis with bootstrap values 1000. 30 protein sequences (Table S2) were divided into five classes based on previous studies, including the CRC class, INO class, YAB2 class, YAB5 class, and YAB3 class (Fig. 1). Based on the phylogenetic tree, the YAB2 class could be divided into two subgroups. The YAB3 class had the largest number of members (11), including six Moso bamboo members. Only one member *AtYABBY05* was present in the YAB5 class. Within the CRC class, there were two *P. edulis*, one *O. sativa* and two *A. thaliana* YABBY proteins. In the YAB2 class, there was only one *Arabidopsis* YABBY protein sequence in the YAB2-II subgroup, and the YAB2-I subgroup contained six Moso bamboo protein sequences.

### Chromosome localization and collinearity analysis of *PeYABBY genes*

Chromosome localization and gene duplication allow analysis of the evolutionary history of the YABBY gene family. Among YABBY family members, 16 *PeYABBY* genes were randomly distributed on 11 chromosome scaffolds based on the whole-genome GFF annotation file of the *P. edulis* and the location information of the *PeYABBY* genes. All chromosome scaffolds had at least one YABBY gene, but the overall distribution was uneven. One *PeYABBY* gene was present on scaffolds 3, 5, 11, 12, 13 and 23, and two *PeYABBYs* were present on scaffolds 4, 10, 15, 21, and 24.

**Figure 2:**
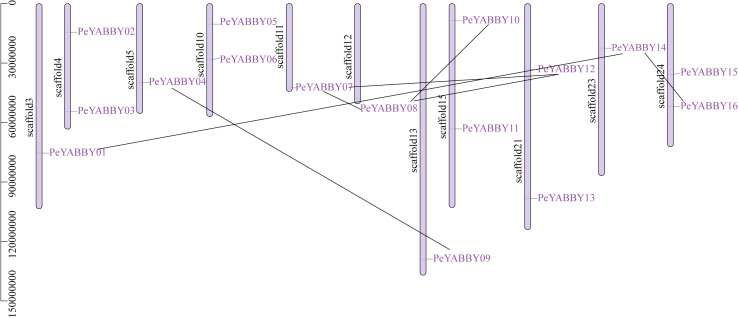
Chromosome location of YABBYgenes in Moso bamboo. Positions of YABBY gene family members on Moso bamboo chromosomes. *PeYABBY* genes are numbered 1-16. The chromosome numbers and gene names are marked adjacent to the chromosome scaffolds and the gene pairs from segmental duplications are linked with black lines.

Intra-specific collinearity analysis was performed to examine YABBY homologous loci relationships and putative gene duplication events. This analysis revealed that many *PeYABBY* genes appeared to have arisen from gene duplications, including *PeYABBY01*/*PeYABBY14*, *PeYABBY04*/*PeYABBY09*, *PeYABBY07*/*PeYABBY08*, *PeYABBY07*/*PeYABBY12*, *PeYABBY08*/*PeYABBY10*, *PeYABBY08*/*PeYABBY12*, and *PeYABBY14*/*PeYABBY16*. In the Moso bamboo gene database, seven homologous *PeYABBY* gene pairs were found on scaffold3, scaffold5, scaffold11, scaffold12, scaffold13, scaffold15, scaffold21, scaffold23, and scaffold24. These results indicated that duplications were responsible for the diversity of the YABBY genes in *P. edulis* (Fig. 2).

**Figure 3:**
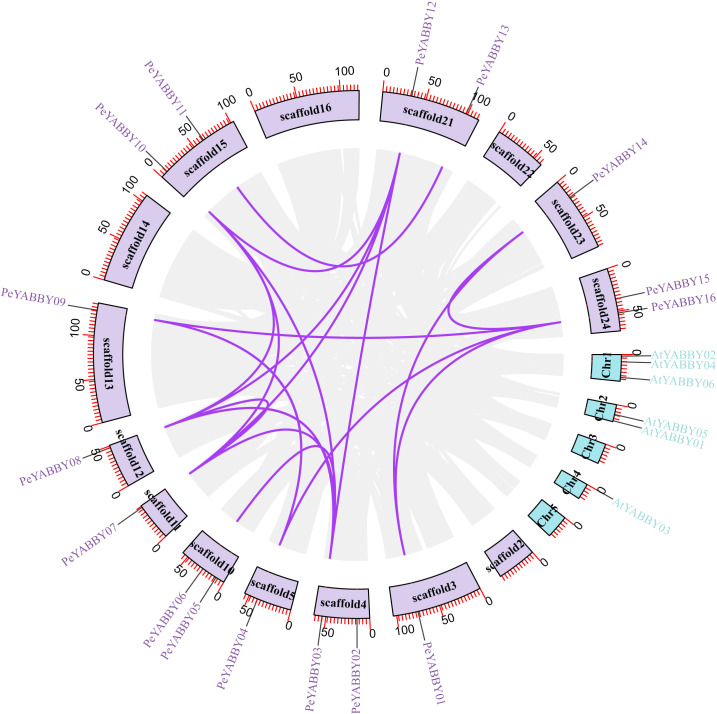
The synteny relationships between Moso bamboo and *Arabidopsis*. Chromosomal distribution and expansion analysis of YABBY genes between two species, the lavender lines indicate the *PeYABBY* gene expansion of Moso bamboo.

**Figure 4:**
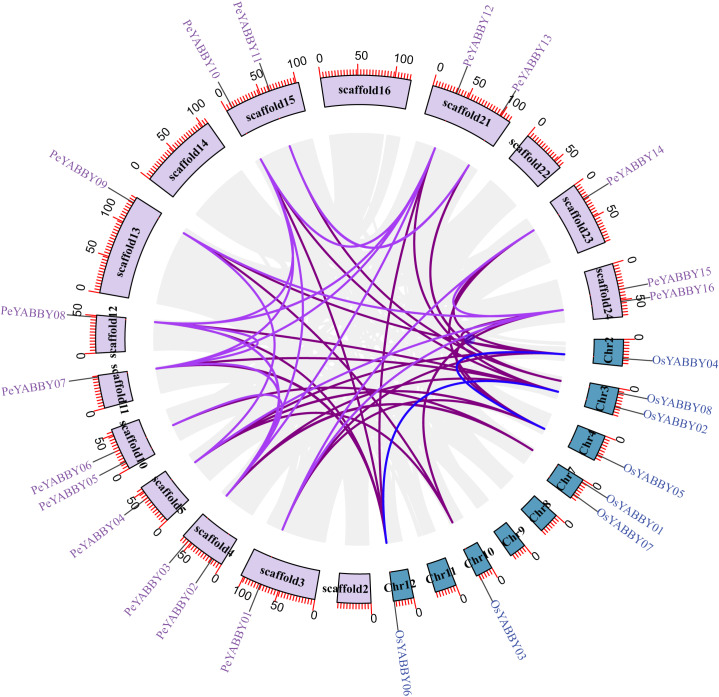
The synteny relationships between Moso bamboo and *O. sativa*. Chromosomal distribution and expansion analysis of YABBY genes in two species. The lavender lines indicate the *PeYABBY* gene expansion of Moso bamboo, while the blue lines indicate the *OsYABBY* gene expansion of *O. sativa*. The modena lines indicate the YABBY genes expansion between Moso bamboo and *O. sativa*.

A collinearity analysis was performed in order to determine the gene duplication relationships in Moso bamboo compared to orthologous genes found in other species. This analysis identified several gene duplications in Moso bamboo relative to rice, but there was no collinearity relationship between Moso bamboo and *A. thaliana*. This gene duplication phenomenon suggested that the Moso bamboo YABBY complement was more closely related to monocotyledons (Figs. 3 and 4).

To further explore the relationship between purifying selection and YABBY family gene duplication and differentiation, the nonsynonymous (Ka), synonymous (Ks), and Ka/Ks substitution ratios were calculated for the duplicated gene pairs in order to estimate the age of the duplication events (Table 2). The Ks values for fragment replication ranged from 0.13 to 0.40. Thus, the inferred discrete time range was from 9.17 million years ago (Mya) to 77.84Mya. The Ka/Ks values of fragment replications were less than 1, indicating that they underwent purifying selection.

### Gene structure, motifs and conserved domains-sequences **of***YABBY***in***P. edulis*

Sixteen genes were identified, which were divided into four subfamilies, including INO (INNER NO OUTER), CRC (CRABS CLAW), YAB3, and YAB2-I. The evolutionary aspects and structural diversity of the *PeYABBY* genes in bamboo were investigated by studying the exon-intron organization. All the *PeYABBYs* contained several introns, and the number of introns varied from 2 to 7 (Fig. S1). Additionally, two genes of the INO subfamily had no untranslated regions (UTR), while the CRC subfamily, which contained two genes (*PeYABBY11* and *PeYABBY13*), contained a UTR at only one end. It was worth noting that the *PeYABBY06* gene of the YAB3 subfamily lacked a UTR. The INO subfamily genes contained the shortest intronic regions and the YAB2-I subfamily genes included the longest intronic regions.

**Table 2:** Ka, Ks, and Ka/Ks calculation and divergent time of the duplicated *PeYABBY* gene pairs.

**Duplicated gene pairs**	**Ka**	**Ks**	**Ka/Ks**	**Purify selection**	**Time (Mya)**
PeYABBY10/PeYABBY08	0.13	0.99	0.13	Yes	76.01
PeYABBY12/PeYABBY07	0.14	1.01	0.14	Yes	77.84
PeYABBY12/PeYABBY08	0.16	0.90	0.17	Yes	69.07
PeYABBY14/PeYABBY01	0.25	0.69	0.36	Yes	53.29
PeYABBY14/PeYABBY16	0.03	0.12	0.27	Yes	9.17
PeYABBY04/PeYABBY09	0.12	0.31	0.40	Yes	23.48
PeYABBY07/PeYABBY08	0.05	0.15	0.30	Yes	11.63

**Notes.**

Ks synonymous substitution rate Ka nonsynonymous substitution rate Mya million years ago

**Figure 5:**
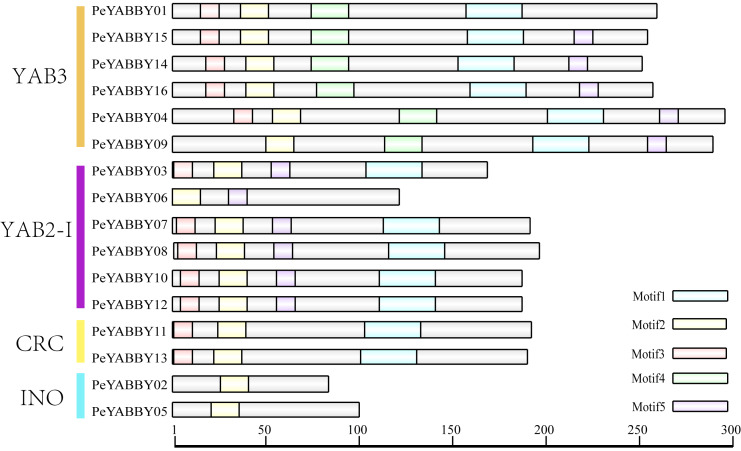
Analysis of YABBY motifs. The proteins of *PeYABBY* are composed of five motifs. The conserved motifs were predicted using with MEME software (https://meme-suite.org/meme/). The bar represents protein sequence length.

**Figure 6:**
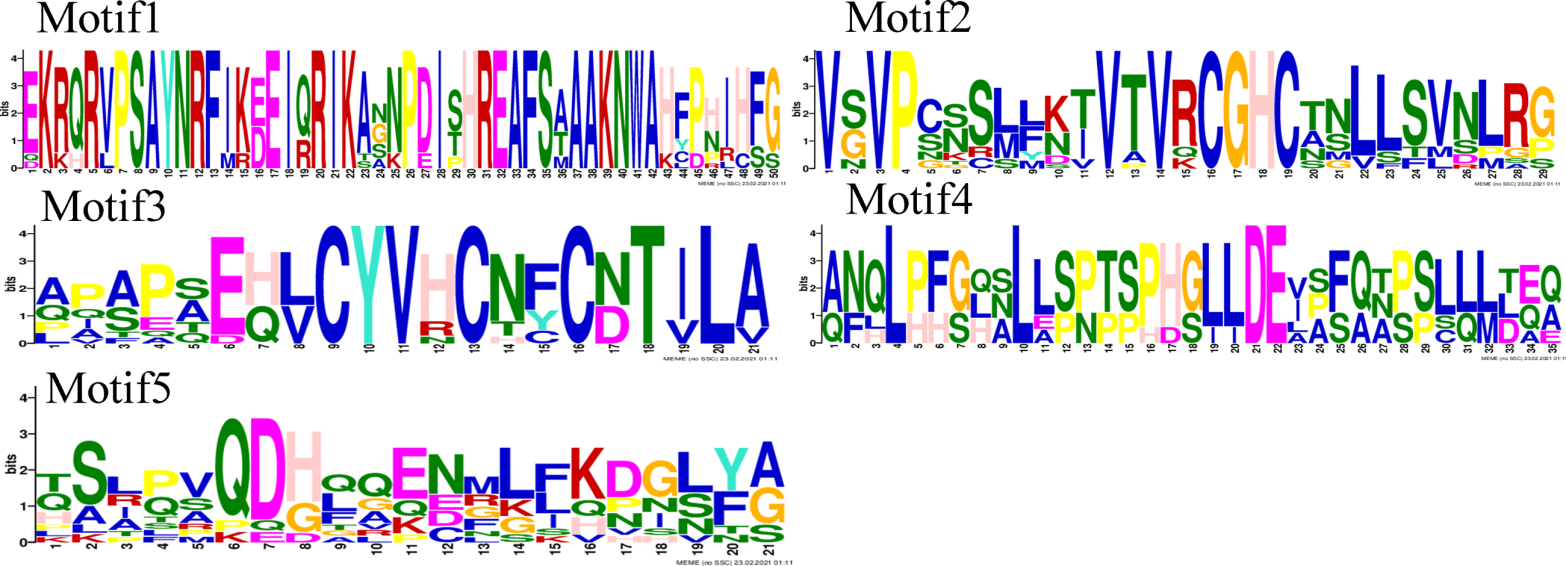
Motif sequence logos. The name of the motifs are shown above the picture.

Motif analysis revealed that the INO subfamily genes only contained one motif. Although each member of the *PeYABBY* gene family contained a variable numbers of motifs, motif 2 was highly conserved (Fig. 5, Fig. S2). *PeBBX06* of the YAB2-I subfamily had only two motifs, which may have resulted from deletion during evolution. The sequence logos of each motif were shown in Fig. 6. Among them, motif 2 and motif 3 contained more cysteine amino acids.

**Figure 7:**
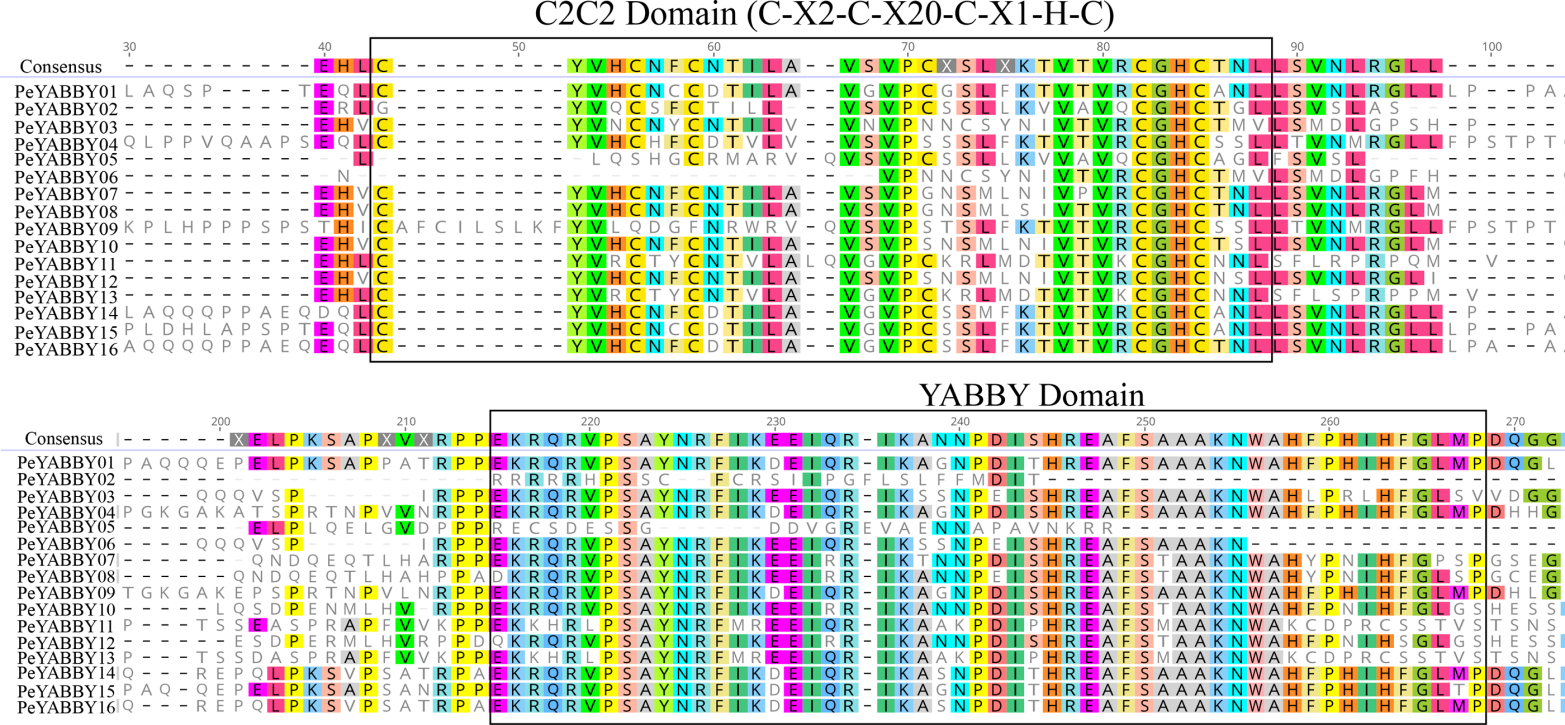
Conserved domains- sequence s analysis of the YABBY proteins. Conservative YABBY structure domain by NCBI CDD software (https://www.ncbi.nlm.nih.gov/Structure/cdd/wrpsb.cgi) compare Pfam database for identification. The main domains are labeled, with the names of the domain displayed at the top of the sequences. On the left side, the name of the YABBY protein is displayed.

In order to analyze the homologous structural domain sequences in the C2-C2 zinc finger and YABBY domains and the degree of conservation of each amino acid residue, multiple sequence alignment analyses were performed. The results was shown in Fig. 7, which revealed a highly conserved C2-C2 zinc finger domain located at the N-terminal end of the protein sequences. In this region, the spatial configuration of the cysteine (C) and histidine (H) residues directly bound to Zn was conserved (C-X2-C-X20-CXL-HC). A 70-aa long YABBY domain was located near the carboxyl terminus, which was similar to the structure of other species’ YABBY proteins. However, the structures of the C2-C2 zinc finger domains for *PeYABBY05*, *PeYABBY06*, and *PeYABBY09* were incomplete.

### Promoter analysis of *PeYABBY* genes

**Figure 8:**
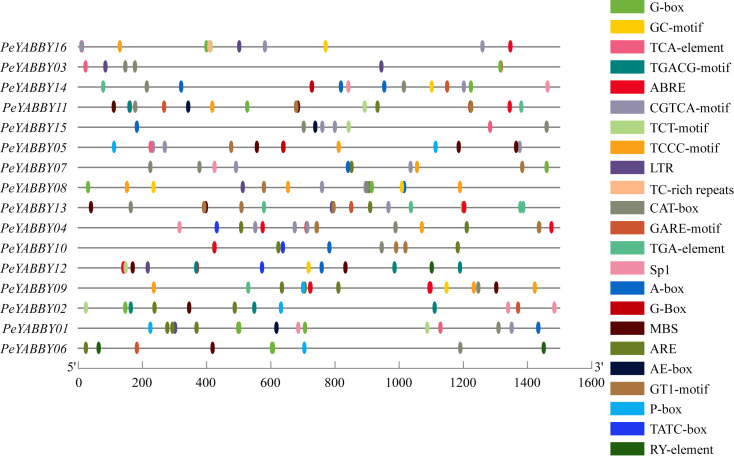
The *cis.*-acting regulatory elements analysis of *PeYABBY* genes. The online analysis program PlantCARE (http://bioinformatics.psb.ugent.be/webtools/plantcare/html/) was used to investigate the 1,500 bp sequences upstream of the ATGs. Different *cis*-acting elements of *Pe YABBY* genes are displayed, with the different colored markers indicating different predicted *cis*-acting elements.

Investigation of promoter region *cis*-acting regulatory elements (CRE) contributes to comprehend the regulation of gene transcription levels. In this study, the number of *cis*-acting elements associated with hormone (including MeJA, ABA, GA, salicylic acid (SA), auxin, and others), light, adversity, and developmental regulation was analyzed by searching the PlantCARE database to identify potential *cis*-acting elements in the 1500 bp upstream promoter region of the *PeYABBY* genes. The results were shown in the Fig. 8. Various hormone-responsive elements and light-responsive elements were found to exist in almost all promoters of *PeYABBY* genes. GA-responsive elements (GAREs), including a GARE-motif, P-box and TATC-box, were predicted to exist in the promoters of *PeYABBY14*, *PeYABBY11*, *PeYABBY13*, *PeYABBY04*, *PeYABBY10*, *PeYABBY12*, *PeYABBY05*, *PeYABBY09*, *PeYABBY02*, *PeYABBY01*, and *PeYABBY06*. GARE-motifs were predicted to be present in the promoters of *PeYABBY14*, *PeYABBY11*, *PeYABBY13*, *PeYABBY02*, and *PeYABBY06*. The TATC-box was predicted to exist in the promoters of *PeYABBY04*, *PeYABBY10*, and *PeYABBY12*. TCA-element, the SA responsiveness element, could be found in the promoter regions of *PeYABBY16*, *PeYABBY03*, *PeYABBY11*, *PeYABBY15*, *PeYABBY05* and *PeYABBY01*. The abiotic stress-responsive TC-rich element was present in *PeYABBY16* gene only, and the seed-regulated growth RY-element was present in the promoters of *PeYABBY12* and *PeYABBY06*. At the same time, anaerobic response elements (ARE), drought-resistant elements (MBS) and low temperature response elements (LTR) were identified as *cis*-acting regulatory elements associated with abiotic stress.

### Expression patterns of the *PeYABBY* genes **in different tissues and organs**

**Figure 9:**
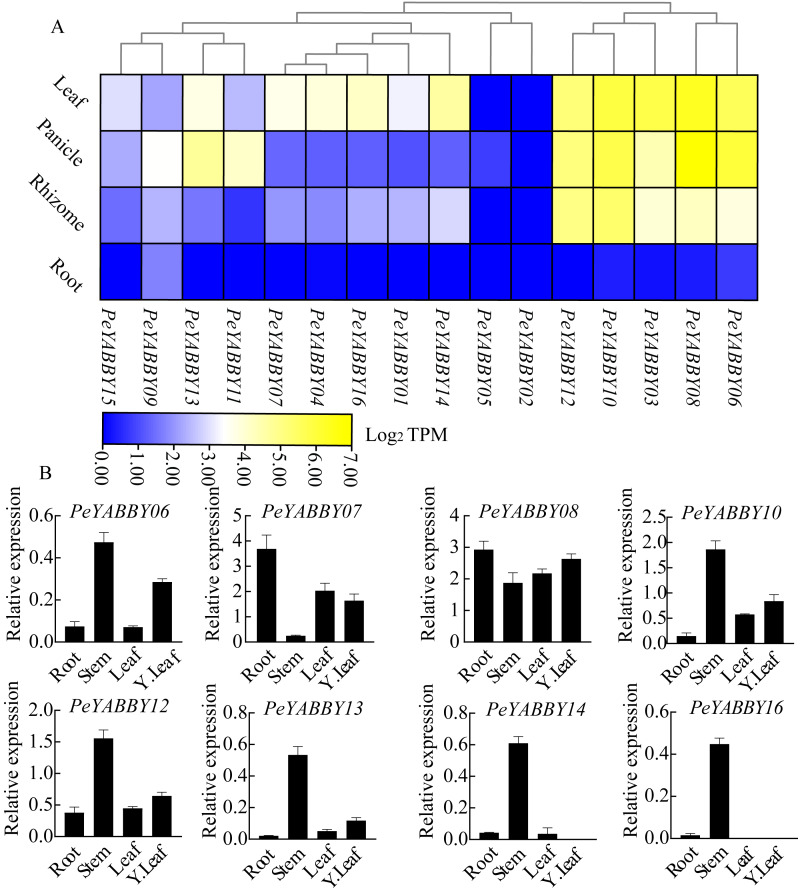
Expression profile cluster analysis of *PeYABBY* genes with differential tissue expression. (A) Heatmap showing relative expression levels of *PeYABBYs* in roots, leaves, panicles, and rhizomes. The values are expressed with log_2_ TPM. (B) Expression of *PeYABBY* s in different tissues. Expression levels were calculated using the 2^ΔCt^ method, this figure shows the eight *PeYABBY* genes with expression levels more than 0.5.

To obtain the expression pattern of the YABBY genes in different tissues of Moso bamboo, we used the relevant transcriptome data available from the Genome Database website to examine the expression patterns of the Moso bamboo YABBY genes. In order to explore the potential function of *PeYABBY* genes in *P. edulis*, the expression levels of *PeYABBYs* in different tissues were examined, including root, rhizome, panicle, and leaf. Expression heatmaps were drawn using TBtools software with log_2_ TPM algorithm. The *PeYABBY* genes were shown to have clear tissue specificity (Fig. 9A, Table S3). Most *PeYABBY* genes had low root expression. *PeYABBY06*, *PeYABBY08*, *PeYABBY03*, *PeYABBY10*, and *PeYABBY12* were highly expressed in leaves, panicles, and rhizomes despite having very low expression in roots. *PeYABBY09* was significantly expressed in roots, leaves, panicles, and rhizomes, while *PeYABBY02* was not expressed in roots, leaves, panicles, or rhizomes. *PeYABBY11* and *PeYABBY13* were significantly expressed in panicles.

Next, the expression patterns of 16 members of the YABBY gene family in different tissues were then investigated using qRT-PCR. The results showed that *PeYABBY16* were all expressed significantly in stems, but not in leaves, or young leaves. *PeYABBY07* showed low expression in stems but significant expression in other tissues and the expression of *PeYABBY08* was significant in four tissues. The expression patterns of *PeYABBY06*, *PeYABBY10*, *PeYABBY12* and *PeYABBY13* were similar in the four tissues, and it was obvious that the expression was most significant in the stem, followed by the young leaves, and then the tissues of leaves and roots (Fig. 9B). These results indicated that the genes of this family had obvious tissue specificity.

### Responses of *PeYABBY* members under various phytohormonal treatments

**Figure 10:**
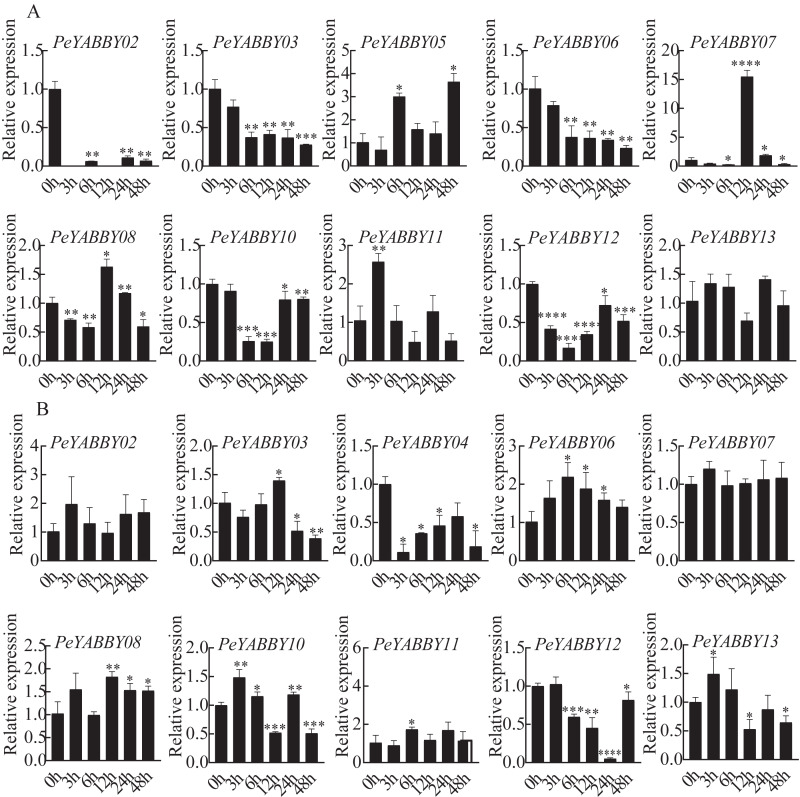
QRT-PCR analyses of *PeYABBY.* genes under phytohormone treatments. (A) The expression patterns of *PeYABBY s* under ABA treatment. (B) The expression patterns of *PeYABBY s* under GA treatment. The concentration of ABA and GA was 100 µM and 50 µM, respectively. A single asterisk shows that the level of gene expression in the treatment group was substantially different from the control group (*t*-test, *p* < 0 .05). There was a much more significant difference (*t*-test, *p* < 0.01) when double asterisks were used. The 2^−ΔΔCt^ method was used to look at the relative expression levels of the target genes. The standard deviations of the means of independent replicates are expressed by error bars.

The presence of predicted *cis*-acting regulatory elements in *PeYABBY* gene promoters indicated that they were likely involved in hormone responses. To test this hypothesis, we applied ABA and GA treatments to Moso bamboo seedlings. qRT-PCR was used to evaluate the relative expression levels of the *PeYABBYs* under phytohormonal treatments. Two genes (*PeYABBY07* and *PeYABBY08*) were significantly up-regulated at 12 h after ABA treatment, while *PeYABBY11* was up-regulated at 3 h after ABA treatment. Some *PeYABBY* genes were significantly down-regulated at each time point following ABA treatment, including *PeYABBY02*, *PeYABBY03*, *PeYABBY06*, *PeYABBY10*, and *PeYABBY12* (Fig. 10A). As shown in Fig. 10B, *PeYABBY06* was significantly up-regulated at each time point following GA treatment. The expression of *PeYABBY13* was down-regulated by GA only at 12 h, 24 h, and 48 h after treatment. In addition, *PeYABBY04* was down-regulated at all time points after GA treatment. Furthermore, *PeYABBY03* was up-regulated at 12 h after GA treatment only but was down-regulated at all other time points following GA treatment. Taken together, these results indicate that YABBY family genes were highly responsive to different hormone treatments.

### Protein interaction network and GO Enrichment

**Figure 11:**
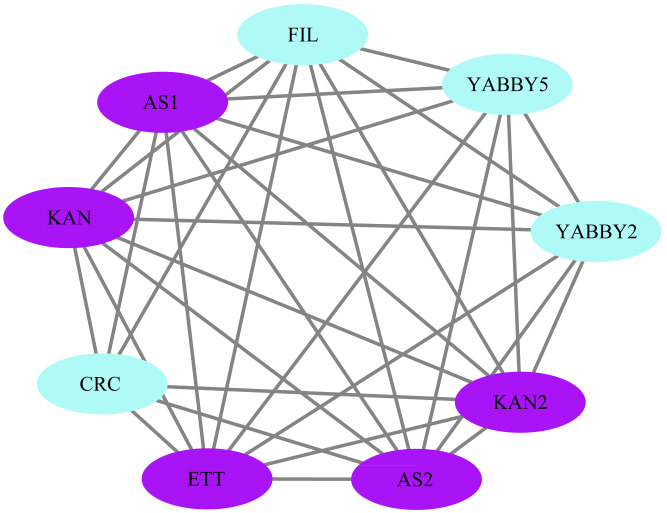
Analysis of protein interaction networks. Using the STRING online database, YABBY genes were selected and used to construct a PPI network. Nodes represent proteins, while black lines indicate interactions between nodes.

The STRING database was used to construct a network of protein relationships in order to better understand the potential functions, signaling, and metabolic interactions of the YABBY proteins (The corresponding gene IDs can be found in Table S5). FIL/YABBY2 and AS2/YABBY5 were predicted to regulate the initiation of embryonic shoot apical meristem (SAM) development. CRC/KAN and KAN2 were predicted to coordinate the regulation of carpel development. In addition, CRC was predicted to interact with other unspecified proteins to regulate leaf polarity and the formation of floral organs (Fig. 11).

**Figure 12:**
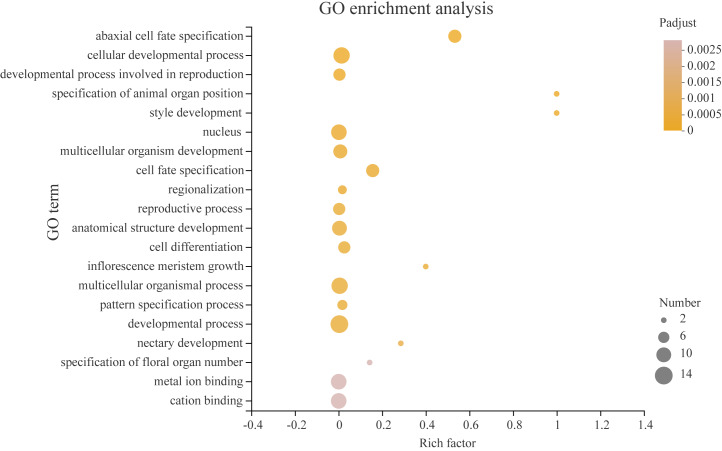
Gene ontology (GO) enrichment analysis. Comparative analysis using the GO database. The horizontal axis indicates the enrichment factor, and the size of the circle indicates the number of genes annotated with a given GO term. Padjust less than 0.05 was considered to be significant enrichment. The analysis results show the top 20 significantly enriched information for functional annotations.

Transcription factors play a critical role in the growth and development of plants. In order to better understand what processes *PeYABBY* genes were involved in, we performed GO enrichment analysis (Fig. 12, Table S6). In total, 8 of the *PeYABBY* genes were involved in abaxial cell fate specification (GO: 0010158), 10 *PeYABBY* genes regulated anatomical structure development (GO: 0048856), and 14 *PeYABBY* genes were involved in multicellular biological processes (GO: 0032502). At the CC level, it could be seen that the *PeYABBY* family genes of Moso bamboo were mainly involved in forming the nucleus (GO: 0005634), and their molecular functions were mainly focused on binding metal and cations (GO: 0046872 and GO: 0043169).

### Homologous modeling of protein structures of *PeYABBY***genes**

**Figure 13:**
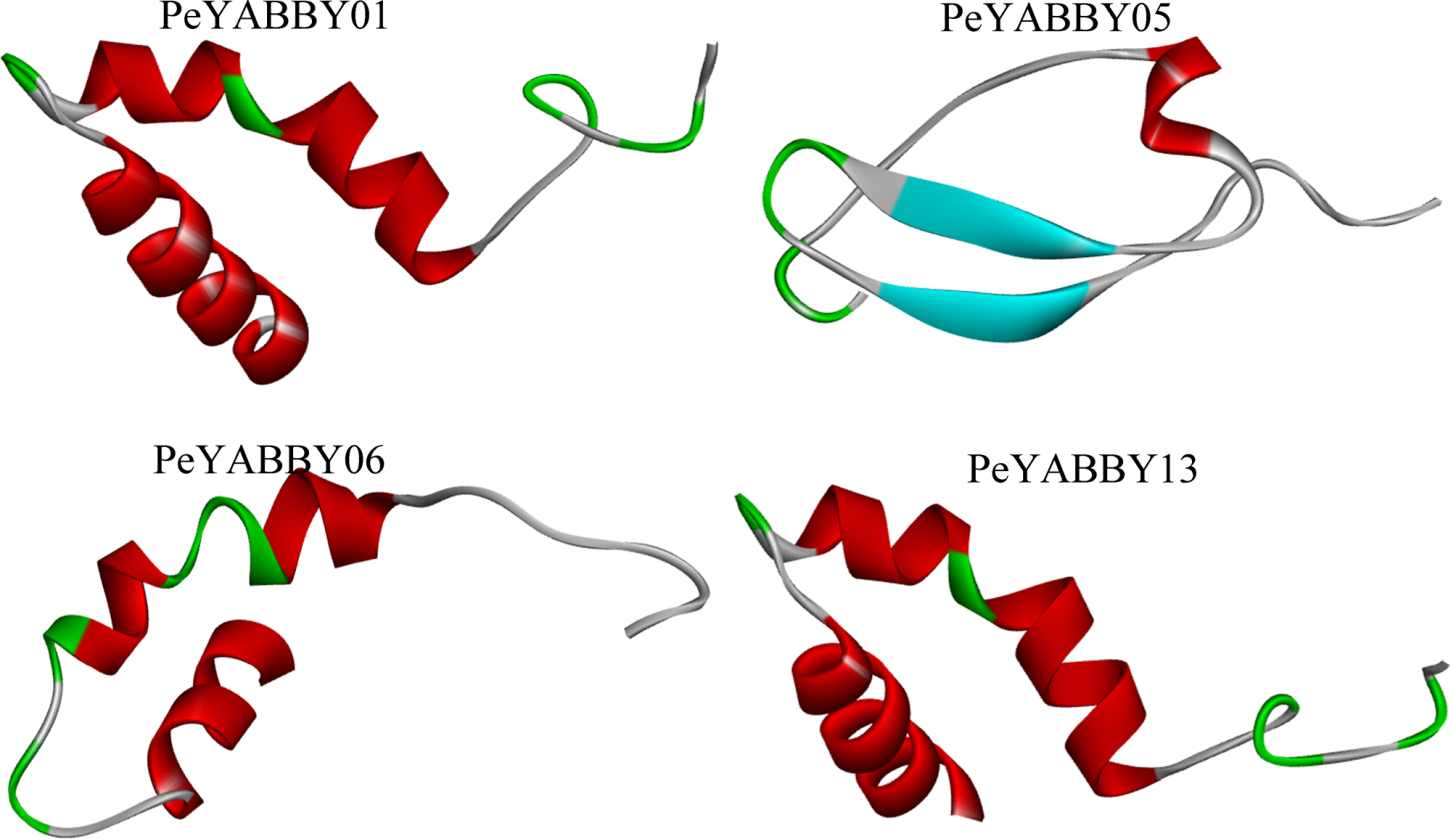
Tertiary structure analysis of YABBY proteins. The diagram shows the tertiary structure of four YABBY proteins from four subfamilies. The protein of *PeYABBY01* belongs to the YAB3 subfamily, while the protein of *PeYABBY05* belongs to the INO subfamily, *PeYABBY06* belongs to the YAB2-I subfamily, and *PeYABBY11* belongs to the CRC subfamily.

The secondary structure of the YABBY family of proteins in Moso bamboo consists mainly of irregular coiling, while *α*-helix, extended chain (Ee), and *β*-turn angle are scattered throughout the protein. Tertiary structure analysis revealed that the subfamily members were similar in structure, with two *α*-helices and one loop (See the S3.Fig). There were also differences in the structure of different subfamily genes. For example, *PeYABBY05* had a *β*-fold structure, while the structure of *PeYABBY11* consisted mainly of three *α*-helices. (Fig. 13).

## Discussion

The YABBY family of genes, with typical zinc finger and YABBY conserved domains, is a plant-specific transcription factor involved in the growth and development of flowers, seeds, leaves, and buds (Shamimuzzaman & Vodkin, 2013; Bartholmes, Hidalgo & Gleissberg, 2012; Cong, Barrero & Tanksley, 2008; Alvarez & Smyth, 1999). In addition, it was shown that YABBY was bifunctional transcription factor as a repressor or activator (Stahle et al., 2009). *MSYABBY5* negatively regulates triticale biosynthetic processes and activates *MSWRKY75* in spearmint (Wang et al., 2016). The number of YABBY genes varies among species, with six members present in *A. thaliana* (Finet et al., 2016; Villanueva et al., 1999), eight YABBY genes identified in rice (Eckardt, 2010; Toriba et al., 2007), seven in grapes, seventeen in soybeans (*Glycine max*) and nine in pineapple (*Ananas comosus* L.) (Zhang et al., 2019; Zhao et al., 2017b; Li et al., 2019). Despite their importance in growth and development, there has been no report examining the YABBY gene family in *P. edulis*. In this study, we conducted a comprehensive analysis of the YABBY genes of *P. edulis*, leading to a deeper understanding of their functional characteristics.

### Identification and classification of YABBY gene families in *P. edulis*

We identified 16 YABBY genes in Moso bamboo, which encoded proteins ranging from 80 to 300 aa. In order to determine the phylogenetic relationship between YABBY proteins and other species, a phylogenetic tree was constructed, which revealed that no YABBY proteins were found belonging to the YAB5 subfamily, but *PeYABBY* genes belonging to four subfamilies were found, including YAB3, YAB2, CRC, and INO. The monocotyledonous plant species pineapple has also been reported to not contain YAB5-like genes (Li et al., 2019). These genes were all similar in structural composition, indicating that the genetic structure of the YABBY subfamily was highly conserved. The different subfamilies of YABBY have been proposed to play different roles in plant growth and development. *FIL*, *YAB2*, and *YAB3* are all expressed in aboveground lateral organ primordia and specifically determine the fate of stem cells at the distal end of lateral organs. *FIL*, *YAB3*, and *YAB5* show functional redundancy during *Arabidopsis* leaf development and are mainly expressed in nutrient storage tissues (Stahle et al., 2009; Sarojam et al., 2010; Eckardt, 2010), while *CRC* and *INO* are specifically expressed in floral organs (Eckardt, 2010). *CRC* genes mainly regulate the development of carpels and nectaries in *Arabidopsis* (Bowman et al., 1999; Fourquin et al., 2007), and INO genes play an important function during outer integument development in *Arabidopsis* (Yang et al., 2018).

### The YABBY gene family expanded and evolved

In plant growth processes, gene duplication plays an important role, YABBY genes may have undergone functional divergence after duplication, and some may have lost their original function, acquired new function, or retained the original functional delineation (Wang et al., 2014; Xu et al., 2012; Zhao et al., 2017a). In the *PeYABBY* gene family, there were no tandem gene duplications, but seven gene pairs generated via segmental duplications were found. Gene duplication is thought to be a significant contributor to the evolutionary process, and these duplications may have allowed for an expansion of YABBY gene function in plants (Huang et al., 2013). Additionally, multiple homologous pairs of genes were identified between Moso bamboo and *O. sativa*, suggesting that YABBY genes in these two species originated from the same ancestral genes that had differentiated prior to species divergence. However, no gene duplication was found in Moso bamboo and *Arabidopsis*, likely because of the relatively large evolutionary distance between monocotyledons and dicotyledons. The evolutionary tree showed that the YABBY genes of Moso bamboo and the YABBY genes of *O. sativa* were clustered in one branch, again indicating that these species shared a common ancestor which had a similar complement of YABBY genes (Wu et al., 2014).

Ka/Ks ratios can be used to assess whether protein-coding genes have undergone positive selection (Ka/Ks ratio >1), neutral selection (ratio = 1), or purifying selection (ratio <1) (Hurst, 2002). Our analysis showed that the Ka/Ks ratios of the YABBY homologous pairs were less than 1, indicating that purifying selection was primarily impetus for YABBY family during evolution (Yang et al., 2018). The dates of segmental duplication events (9.17–77.84 Mya) indicated that segmental duplication of the *PeYABBY* genes has occurred after the differentiation of monocot-dicot split, and which were observed at 170–235 Mya (Inal et al., 2017; Gupta et al., 2015).

### Functional analysis of the YABBY gene family

Under both biotic and abiotic stresses, *cis*-acting regulatory elements act as molecular switches that are closely related to the regulation of gene expression (Tian et al., 2019). In the YABBY gene family, we found a large variety of *cis*-acting elements, many of which were associated with responses to GA and ABA hormones. Each gene contained hormone-responsive elements, in which *PeYABBY02*, *PeYABBY05*, and *PeYABBY06* contained more GA hormone-responsive elements. The *PeYABBY* genes showed significant differences in gene expression patterns when treated with ABA or GA hormone. Furthermore, the *PeYABBY* genes (*PeYABBY07*, *PeYABBY08*, *PeYABBY10*, *PeYABBY12*, *PeYABBY03* and *PeYABBY06*) that was responsive to GA was found to be a close homolog of *OsYABBY1*, which has previously been implicated in the feedback regulation of GA metabolism in *O. sativa* (Toriba et al., 2007; Dai et al., 2007b). In total, ten *PeYABBY* genes responded to GA/ABA hormone treatment, suggesting that this gene family plays an important role in hormone regulation.

YABBY genes have previously been proven to play an essential role in plant growth and organ development, with a particular enrichment for roles in the development of trophic organs (Kumaran, Bowman & Sundaresan, 2002; Ha, Jun & Fletcher, 2010; Soundararajan et al., 2019; Zhao et al., 2020). Heatmap expression analysis revealed that the *PeYABBY* genes were expressed at a lower level in the roots, *PeYABBY06*, *PeYABBY10* and *PeYABBY12* genes were expressed at high levels in leaves, rhizomes and panicles, which suggesting that these genes might be involved in different developmental processes, which was consistent with the study by Dai et al. (2007a).

The YABBY genes have tissue expression specificity, it was shown that two genome members, CRABS CLAW and INNER NO OUTER, were expressed only in the floral organs. In contrast, members of the FILAMENTOUS FLOWER, YABBY2, and YABBY5 genomes were also expressed in the leaves (Eckardt, 2010; Bartholmes, Hidalgo & Gleissberg, 2012). The rice *OsYAB1* protein is highly homologous to YAB2 and YAB5 in *Arabidopsis*. These genes have flower-specific expression, and *OsYAB1* has been shown to play an important role in the development of rice meristematic tissues, as well as the developmental processes of stamens and carpels (Jang et al., 2004). A *TOB1* gene encoding the YABBY protein, which is closely related to *Arabidopsis* FIL in rice, is expressed in the lateral organ progenitor but is not detectable in meristematic tissues. Wang et al. (2009) also found that *LiYAB1* was more strongly expressed in lily carpels and weakly expressed in leaves. The qRT-PCR expression data revealed that there were two genes (*PeYABBY14*, *PeYABBY16*) that were not expressed in the young leaves, while all eight *PeYABBY* genes were expressed in the stems. Therefore, analysis of the expression patterns of these *PeYABBY* genes indicated that many had tissue-specific expression patterns.

GO (Gene Ontology) analysis of YABBY proteins showed that they were involved in numerous developmental processes and show a resistance to environmental stress (Buttar et al., 2020). Kelley, Skinner & Gasser (2009) reviewed that two major polarity determinants were the KANADI and YABBY gene families, that the KANADI and YABBY gene families are two major polarity determineors, which are thought to be involved in integument development. Two KAN members (KAN1 and KAN2) of KANADI were found to be involved in the development of the outer integument together with the YABBY gene family member INO and were essential regulators of lamellar extension of the outer tegument (Simon et al., 2012). The PPI network prediction revealed that YABBY interacted with KANADI family proteins to co-regulate plant growth and development. Moreover, Boter et al. (2015) has been shown that YABBY interacts with transcriptional repressors (JASMONATE-ZIM DOMAIN [JAZ] proteins) through its N-terminal domain and is involved in the regulation of jasmonate (JA)-related pathways. After JA triggering, the SCF receptor complex (SCFCOI1) degrades JAZ3 and releases YABs, which activate a subset of JA-regulated genes in leaves, leading to anthocyanin accumulation, chlorophyll loss, and reduced bacterial defense (Boter et al., 2015).

An evolutionary theory of the YABBY gene in angiosperms has been reported. Bartholmes, Hidalgo & Gleissberg (2012) proposed that “trophic” YABBY (FIL/YAB3, YAB2 and YAB5) do not form monophyletic branches and that CRC and FIL evolved from a common ancestral gene of which is sister of INO gene. Tertiary homology modeling revealed that the INO subfamily was structurally specific and did not contain the helix-loop-helix structure, such as *PeYABBY5* contained atypical structural *β*-folded lamellaes, which might be due to functional mutations that occurred during evolution.

## Conclusions

In this study, 16 *PeYABBY* genes were identified and systematically analyzed. The results of the study showed that YABBY genes from Moso bamboo could be classified into three different subfamilies, which was different from the classification of other plants. Each sequence had unique sequence features beyond the conserved amino acid structural domain. Chromosomal/segmental duplication, tandem gene duplication, might contribute to the expansion of the YABBY gene family. Transcriptome expression files indicated that the YABBY gene family determined important functions in leaves and panicles of development and growth. And most of the genes could be responsive to hormone treatments. In conclusion, our data provide a basis for clarification of the function and evolution of the YABBY members of the Moso bamboo and provide fundamental information about the YABBY family in the Moso bamboo. And revealed the potential role of *PeYABBYs* in hormone response during the development of *Phyllostachys edulis*.

##  Supplemental Information

10.7717/peerj.11780/supp-1 Supplemental Information 1 Raw data exported from the transcriptome database used for data analysis and to prepare the gene expression patterns shown in Fig. 5 raw data

10.7717/peerj.11780/supp-2 Supplemental Information 2 Gene annotation file for rice The data was downloaded from the rice database and used to construct a phylogenetic tree and to demonstrate interspecific colinearity.

10.7717/peerj.11780/supp-3 Supplemental Information 3 Protein sequence files of rice The data was downloaded from the rice database, and used to construct a phylogenetic tree.

10.7717/peerj.11780/supp-4 Supplemental Information 4 Supplemental Figures and Tables

## Additional Information and Declarations

### Data Availability

The following information was supplied regarding data availability: The raw measurements are available in the Supplemental Files. The RNA-seq data is available at NCBI: ERX082501, ERX082502, ERX082503, ERX082504, ERX082506, ERX082507, ERX082508 and ERX082509
The genomics sequence data of moso bamboo are available at: - the GigaDB Database: Zhao H; Chen C; Fei B; Wang S; Shi C; Liu X; Zhang H; Lou Y; Chen L; Sun H; Zhou X; Wang S; Zhang C; Xu H; Li L; Yang Y; Wei Y; Yang W; Gao Q; Yang H; Gao Z; Zhao S; Jiang Z (2018): Supporting data for ”Chromosome-level reference genome and alternative splicing atlas of moso bamboo (Phyllostachys edulis)” GigaScience Database. http://dx.doi.org/10.5524/100498. - Arabidopsis Information Resource, version 10 (TAIR 10) (https://www.arabidopsis.org/download/index-auto.jsp?dir=%2Fdownload_files%2FGenes%2FTAIR10_genome_release); - Pseudomolecule Download Site webpage in RGAP 7 database (Rice Genome Annotation Project, http://rice.plantbiology.msu.edu/annotation_pseudo_current.shtml).
