# Supplementary material for: Genome-wide identification and analysis of the YABBY gene family in Moso Bamboo (Phyllostachys edulis (Carrière) J. Houz)
Source: PeerJ. 2021 Jul 22;9:e11780. doi: 10.7717/peerj.11780 (PMC8310622; doi:10.7717/peerj.11780)
Supplement: Supplemental Information 4 [file peerj-09-11780-s004.docx]

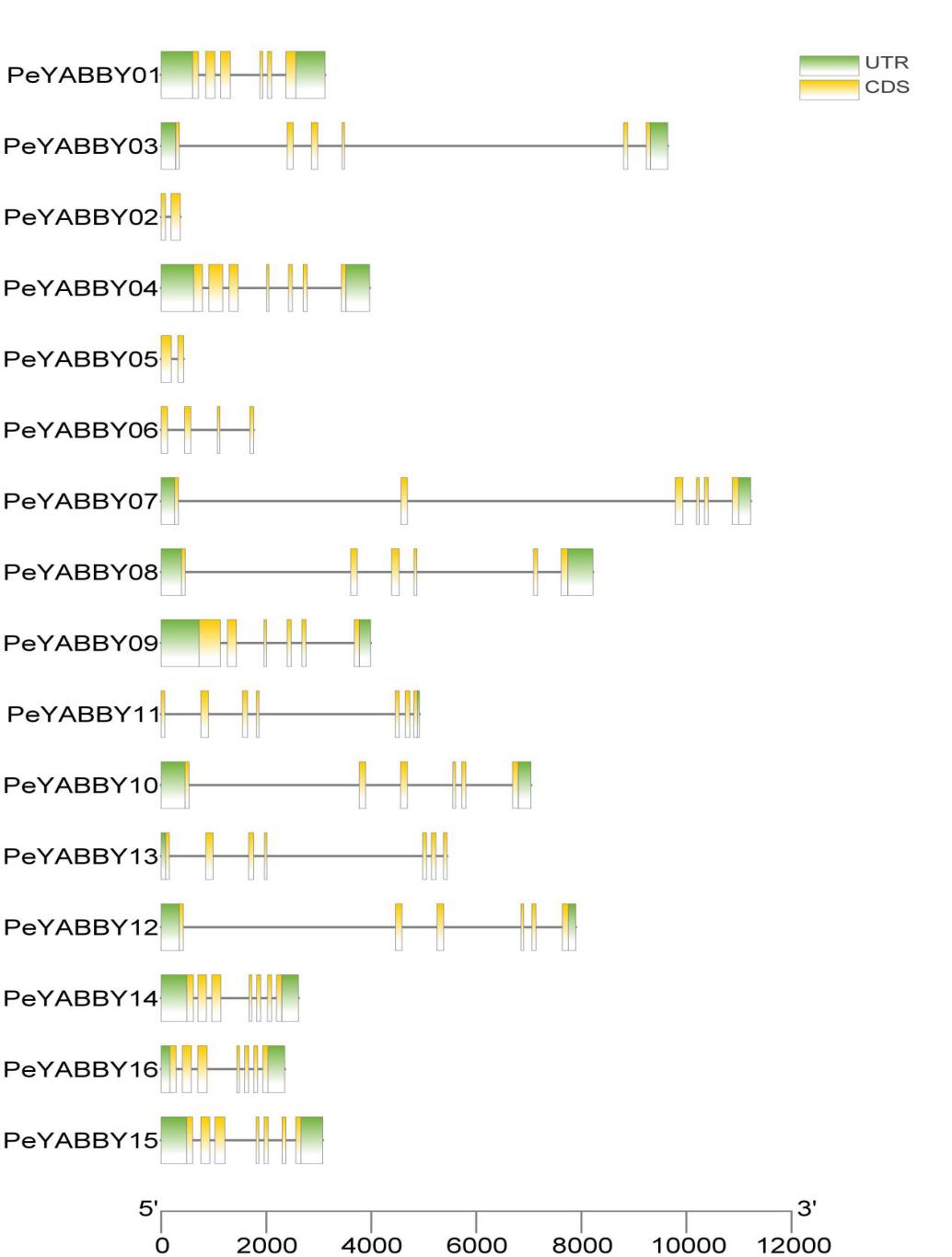


**S1 Figure Analysis of gene structure of *PeYABBYs.*** The scale indicates the length of the nucleotide sequence.


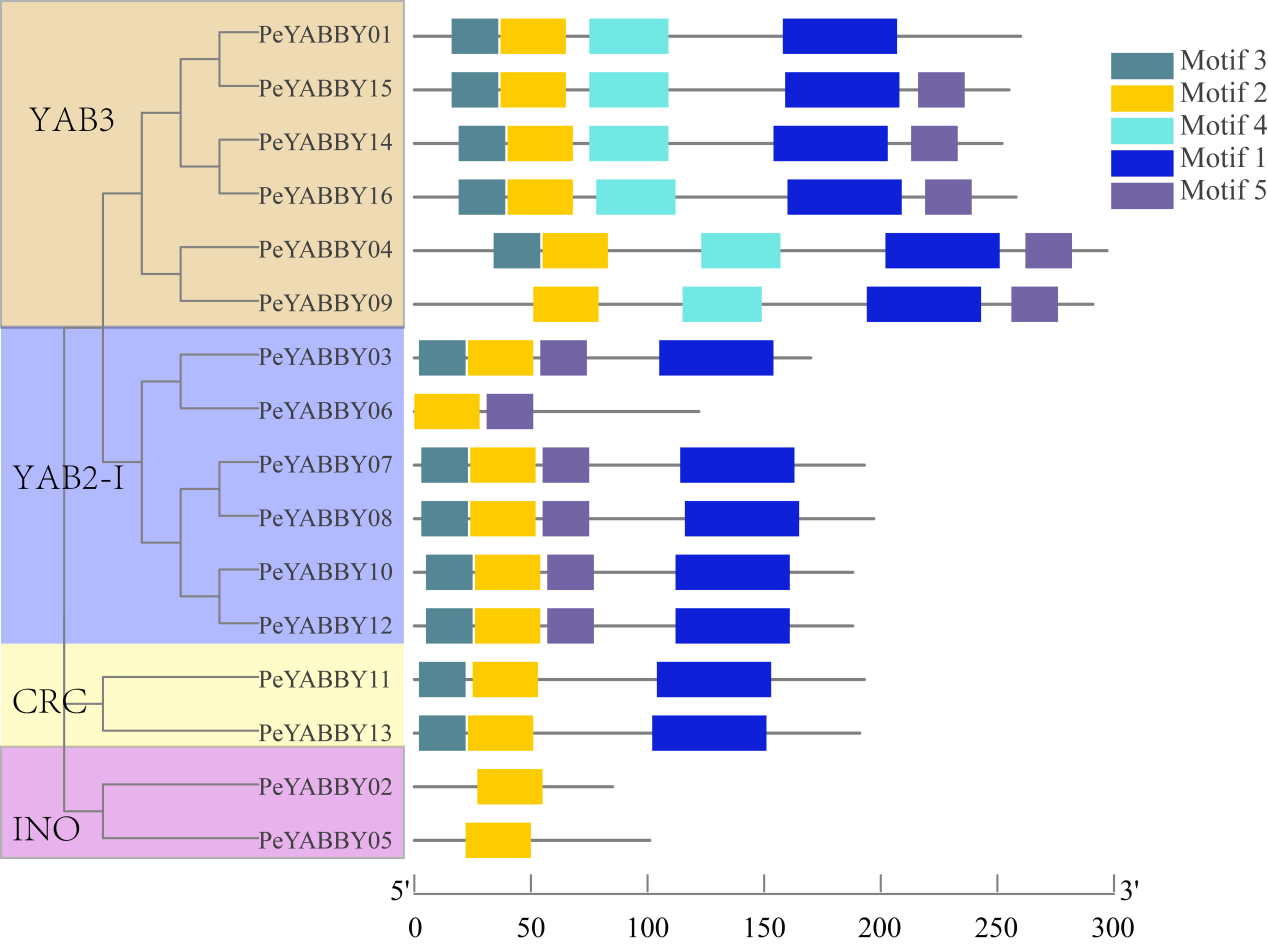


**S2 Figure Analysis of motif of PeYABBY sequences.** The scale indicates the length of the protein sequence.


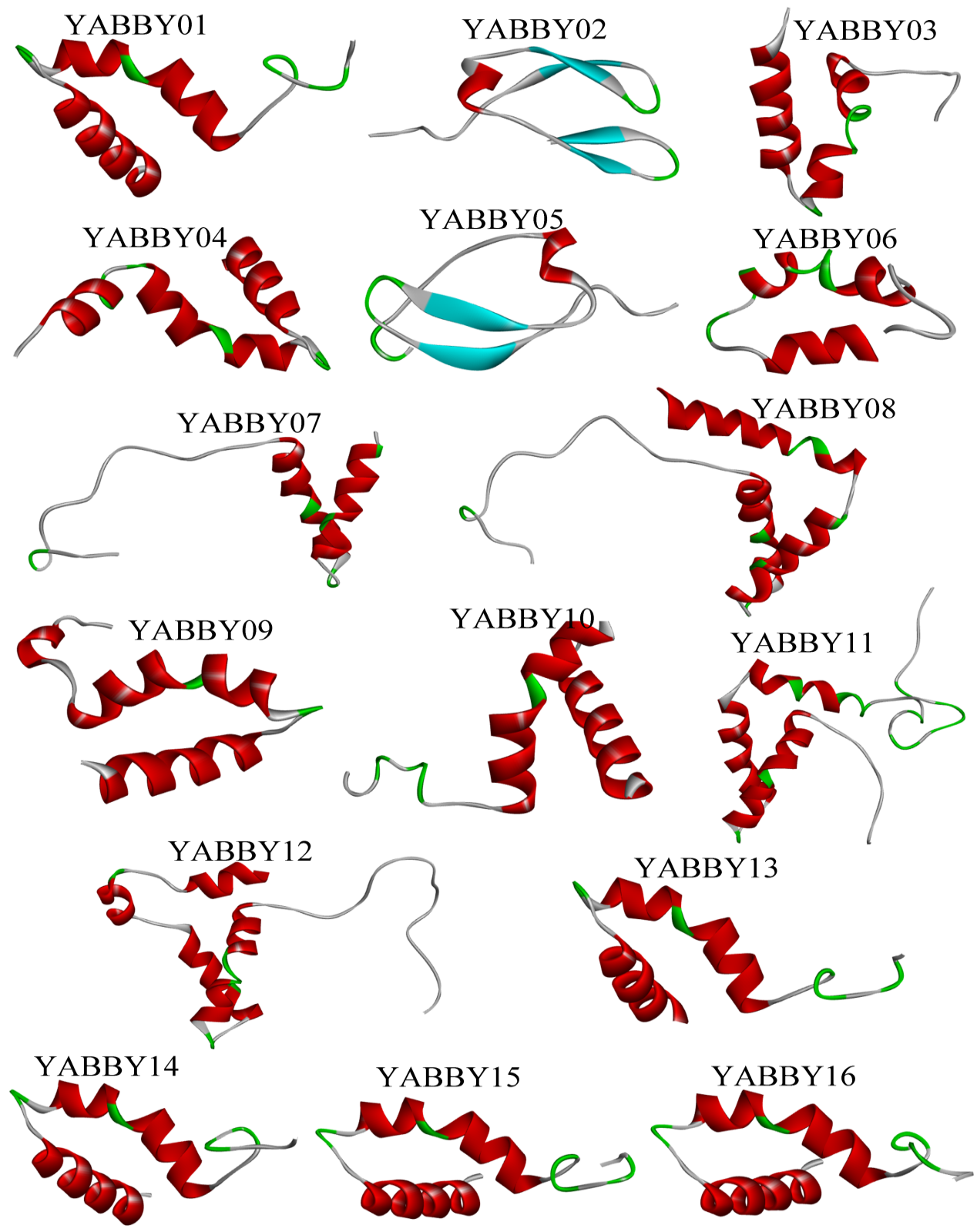


**S3 Figure The tertiary model building of *PeYABBY* genes**

**Table S1 Secondary structure prediction of PeYABBY protein sequence**

| ID rename | Alpha helix | Extended strand | Beta turn | Random coil |
| --- | --- | --- | --- | --- |
| PeYABBY01 | 27.69% | 11.92% | 5.00% | 55.38% |
| PeYABBY03 | 20.00% | 18.24% | 3.53% | 58.24% |
| PeYABBY02 | 18.82% | 28.24% | 12.94% | 40.00% |
| PeYABBY04 | 24.24% | 15.82% | 5.39% | 54.55% |
| PeYABBY05 | 26.73% | 18.81% | 5.94% | 48.51% |
| PeYABBY06 | 24.59% | 11.48% | 4.10% | 59.84% |
| PeYABBY07 | 24.87% | 17.62% | 3.63% | 53.89% |
| PeYABBY08 | 24.87% | 17.26% | 6.09% | 51.78% |
| PeYABBY09 | 23.71% | 18.56% | 4.81% | 52.92% |
| PeYABBY11 | 20.73% | 16.58% | 7.25% | 55.44% |
| PeYABBY10 | 23.40% | 17.55% | 5.32% | 53.72% |
| PeYABBY13 | 21.47% | 14.66% | 4.19% | 59.69% |
| PeYABBY12 | 22.87% | 17.55% | 3.72% | 55.85% |
| PeYABBY14 | 23.41% | 11.90% | 3.17% | 61.51% |
| PeYABBY16 | 28.68% | 13.95% | 5.43% | 51.94% |
| PeYABBY15 | 23.53% | 11.37% | 2.75% | 62.35% |

**Table S2 Protein sequences of the evolutionary tree**

| ID | ID Rename | Sequences |
| --- | --- | --- |
| LOC_Os02g42950.1 | OsYABBY04 | MSSSSSSSAVFPLDHLAAPSPTEQLCYVHCNCCDTILAVGVPCSSLFKTVTVRCGHCANLLSVNLRGLLLPAPAPAPANQLHFGPSLLSPTSPHGLLDEVAFQTPSLLMEQAASASLSSITGRSSSSCASNAPAMQMPPAKPVQQEPELPKNAPASANRPPEKRQRVPSAYNRFIKDEIQRIKAGNPDISHREAFSAAAKNWAHFPHIHFGLMPDQGFKKTFKPQDGSEDILLKDSLYAAAAAAAAAAANMGVTPF |
| LOC_Os03g11600.1 | OsYABBY08 | MDLVSPSEHLCYVRCTYCNTVLALQVGVPCKRLMDTVTVKCGHCNNLSFLSPRPPMVQPLSPTDHPLGPFQGPCTDCRRNQPLPLVSPTSNEGSPRAPFVVKPPEKKHRLPSAYNRFMREEIQRIKAAKPDIPHREAFSMAAKNWAKCDPRCSSTVSTSNSNPEPRVVAAPIPHQERANEQVVESFDIFKQMERSG |
| LOC_Os03g44710.2 | OsYABBY02 | MSAQIVPAPEHVCYVHCNFCNTIFAVSVPSNSMLNIVTVRCGHCTSLLSVNLRGLVQALPAEDHLQDNLKMHNMSFRENYSEYGSSSRYGRVPMMFSKNDTEHMLHVRPPEKRQRVPSAYNRFIKEEIRRIKANNPDISHREAFSTAAKNWAHFPNIHFGLGSHESSKKLDEAIGAPSPQKVQRLY |
| LOC_Os04g45330.1 | OsYABBY05 | MMSSAPETFSLDHLSQHQQQQPPPLAEQEQLCYVHCNFCDTILAVGVPCSSLFKTVTVRCGHCANLLSVNLRGLLLPAAASTANQLPFGQALLSPTSPHGLLDEVPSFQAPASLMTEQASPNVSSITSSNSSCANNAPATSMASAANKATQREPQQPKNAPSANRTSEKRQRVPSAYNRFIKDEIQRIKASNPDITHREAFSAAAKNWAHFPHIHFGLMPDQGLKKTGIQSQDGAGECMLFKDGLYAAAAAAAAATAASSMGVTPF |
| LOC_Os07g06620.2 | OsYABBY01 | MSVQFTSEHVCYVNCNYCNTILVVNVPNNCSYNIVTVRCGHCTMVLSMDLAPFHQARTVQDHQVQNRGFQGNNFGSYDIASRNQRTSTAMYPMPTSQQQVSPIRPPEKRQRVPSAYNRFIKEEIQRIKTSNPEISHREAFSAAAKNRRRRRRRRRQQLIAAARPAGRPPIAGVHAGARRRPVENARAGGTTTIYYYRVCPSLFAA |
| LOC_Os07g38410.1 | OsYABBY07 | MSSAARHHCSGLRERLGCVQCSFCATVLLVSVPCSSVLRVVAVQCGHCSGILSAVNLPPSPVSASIELTPQELDAGPPPGEYSDESSGDDREGRDAEDDAPAPAAAAVANKPPGRKQRTPSAYNCFVKEEIKRIKSMEPNITHKQAFSTAAKNWAHLPRIQQKRGRDSC |
| LOC_Os10g36420.1 | OsYABBY03 | MSSSSSSSASSAAAAAFRPAVVQREQQVVEEKFPAAAAAMREMVLPPVAAAAADSEQEQLCYVHCHYCDTVLVVSVPSSSLFETVTVRCGHCSSLLTVNMRGLLLPTTAAAAPPPPPPPPPPPPPPAAHFPHSLNLAPANPPHHHSLLDEISTANSPTQLLLEQHGLGGLMASAASCRNNNSPAAAAAPPPPTSQGKAAAKEPSPRTNTAVINRPPEKRQRVPSAYNRFIKDEIQRIKAGNPDISHREAFSAAAKNWAHFPHIHFGLMPDHQGLKKTSLLPQDHQRKDGLLKEGLYAAAAAAAAAANMGVAPY |
| LOC_Os12g42610.1 | OsYABBY06 | MSAQIAPAEQVCYVHCNFCNTILAVSVPGNSMLNIVTVRCGHCTNLLSVNLRGLMHSAPALQDHHHHHLQESGLSGCFRDQSGYPEFGFSAASSSSKLRLPPAAAAMVSYSQQNQQLEQALHARPPEKRQRVPSAYNRFIKEEIRRIKANNPDISHREAFSTAAKNWAHYPNIHFGLSPGHEGGKKLVDVDPIPTAPSSKKIQGFYS |
| AT1G08465.1 | AtYABBY02 | MSVDFSSERVCYVHCSFCTTILAVSVPYASLFTLVTVRCGHCTNLLSLNIGVSLHQTSAPPIHQDLQPHRQHTTSLVTRKDCASSSRSTNNLSENIDREAPRMPPIRPPEKRQRVPSAYNRFIKEEIQRIKACNPEISHREAFSTAAKNWAHFPHIHFGLKLDGNKKGKQLDQSVAGQKSNGYY |
| AT1G69180.1 | AtTABBY06(AtCRC) | MNLEEKPTMTASRASPQAEHLYYVRCSICNTILAVGIPLKRMLDTVTVKCGHCGNLSFLTTTPPLQGHVSLTLQMQSFGGSDYKKGSSSSSSSSTSSDQPPSPSPPFVVKPPEKKQRLPSAYNRFMRDEIQRIKSANPEIPHREAFSAAAKNWAKYIPNSPTSITSGGHNMIHGLGFGEKK |
| AT1G23420.1 | AtYABBY04 | MTKLPNMTTTLNHLFDLPGQICHVQCGFCTTILLVSVPFTSLSMVVTVRCGHCTSLLSVNLMKASFIPLHLLASLSHLDETGKEEVAATDGVEEEAWKVNQEKENSPTTLVSSSDNEDEDVSRVYQVVNKPPEKRQRAPSAYNCFIKEEIRRLKAQNPSMAHKEAFSLAAKNWAHFPPAHNKRAASDQCFCEEDNNAILPCNVFEDHEESNNGFRERKAQRHSIWGKSPFE |
| AT2G26580.1 | AtYABBY05 | MANSVMATEQLCYIPCNFCNIILAVNVPCSSLFDIVTVRCGHCTNLWSVNMAAALQSLSRPNFQATNYAVPEYGSSSRSHTKIPSRISTRTITEQRIVNRPPEKRQRVPSAYNQFIKEEIQRIKANNPDISHREAFSTAAKNWAHFPHIHFGLMLESNKQAKIA |
| AT2G45190.1 | AtYABBY01 | MSMSSMSSPSSAVCSPDHFSPSDHLCYVQCNFCQTILAVNVPYTSLFKTVTVRCGCCTNLLSVNMRSYVLPASNQLQLQLGPHSYFNPQDILEELRDAPSNMNMMMMNQHPTMNDIPSFMDLHQQHEIPKAPPVNRPPEKRQRVPSAYNRFIKEEIQRIKAGNPDISHREAFSAAAKNWAHFPHIHFGLVPDNQPVKKTNMPQQEGEDNMVMKEGFYAPAAANVGVTPY |
| AT4G00180.1 | AtYABBY03 | MSSMSMSSSSAPAFPPDHFSSTDQLCYVHCSFCDTVLAVSVPPSSLFKTVTVRCGHCSNLLSVTVSMRALLLPSVSNLGHSFLPPPPPPPPPNLLEEMRSGGQNINMNMMMSHHASAHHPNEHLVMATRNGRSVDHLQEMPRPPPANRPPEKRQRVPSAYNRFIKEEIQRIKAGNPDISHREAFSAAAKNWAHFPHIHFGLMADHPPTKKANVRQQEGEDGMMGREGFYGSAANVGVAHN |
| PH02Gene42615.t2 | PeYABBY01 | MSSSSSSSAAFPLDHLAQSPTEQLCYVHCNCCDTILAVGVPCGSLFKTVTVRCGHCANLLSVNLRGLLLPPAAPPANQLHFGHSLLSPTSPHGLLDEVAFQTSSLLMDQASATLSGITGRSNSSCASNVPAMPMPAAKPAQQQEPELPKSAPPATRPPEKRQRVPSAYNRFIKDEIQRIKAGNPDITHREAFSAAAKNWAHFPHIHFGLMPDQGLRKTVKTQVIKIPRKHMGYLNPNLSLHLEILSNKSEFRARSNTILL |
| PH02Gene08310.t1 | PeYABBY03 | MSAQFASEHVCYVNCNYCNTILVVNVPNNCSYNIVTVRCGHCTMVLSMDLGPSHPARSAQDHQAQNRGFQPNNFGSYESASRNQRTTAMYPMPNNQQQVSPIRPPEKRQRVPSAYNRFIKEEIQRIKSSNPEISHREAFSAAAKNWAHLPRLHFGLSVVDGGGGGGGSSC |
| PH02Gene41238.t1 | PeYABBY02 | MASVRHPWFGLLERLGYVQCSFCTILLVSVPCSSLLKVVAVQCGHCTGLLSVSLASPRRRRRHPSSCFCRSIIPGFLSLFFMDIT |
| PH02Gene30423.t1 | PeYABBY04 | MSSTSSAASAAAAFSPEPQLAENQLPAALQLPPVQAAPSEQLCYVHCHFCDTVLVVSVPSSSLFKTVTVRCGHCSSLLTVNMRGLLFPSTPTGTAATTAAVAVTTTTTTAPPPPAAAAQGQHGQFHLPHSLNLEPNPPHHSLLDEISSANPSLQLLEQHGLGGLIAGAGRTTAPPPPPPPAAPGKGAKATSPRTNPVVNRPPEKRQRVPSAYNRFIKDEIQRIKAGNPDISHREAFSAAAKNWAHFPHIHFGLMPDHHGLKKTSLLPQDHQRKDGLLKDGLYAAAAAAAANMGVAPY |
| PH02Gene17872.t1 | PeYABBY05 | MAMCQPASALLQSHGCRMARVQVSVPCSSLLKVVAVQCGHCAGLFSVSLSSQQPPPPVSVELPLQELGVDPPPRECSDESSGDDVGREVAENNAPAVNKRR |
| PH02Gene42853.t1 | PeYABBY06 | VNVPNNCSYNIVTVRCGHCTMVLSMDLGPFHQARTAQDHQVPNRGFQPNNFGSYESASRNQRTTAMYPVSNNQQQVSPIRPPEKRQRVPSAYNRFIKEEIQRIKSSNPEISHREAFSAAAKN |
| PH02Gene18063.t1 | PeYABBY07 | MSTQIAPAEHVCYVHCNFCNTILAVSVPGNSMLNIVPVRCGHCTNLLSVNLRGLMHSAPVQDHLQENLSKVHSVNGFRDQSGHSEFGSSSSKFRLPMMYSAQNDQEQTLHARPPEKRQRVPSAYNRFIKEEIRRIKTNNPDISHREAFSTAAKNWAHYPNIHFGPSPGSEGGKKLVDDAIAAPAPKKIQRFYS |
| PH02Gene21516.t2 | PeYABBY08 | MSAQITQAEHVCYVHCNFCNTILAVSVPGNSMLSIVTVRCGHCTNLLSVNLRGLMHSAPVQDHLQQENLSKVHSTSGFRDQSGHSELGSSSSRFGLPMMYSAQNDQEQTLHAHPPADKRQRVPSAYNRFIKEEIRRIKANNPEISHREAFSTAAKNWAHYPNIHFGLSPGCEGGGKKHVDEAIATAPAPKKIQGFYS |
| PH02Gene35716.t2 | PeYABBY09 | MCTATSATPSSSSVIKPLHPPPSPSTHICAFCILSLKFYVLQDGFNRWRVQVSVPSTSLFKTVTVRCGHCSSLLTVNMRGLLFPSTPTSTAATAPAIAVTTATAPPPPATGAQHGQFLLPHSLNLAPNPPPDSLLDEISSANSSLQLLEQHGLGGLIAGVGNTTAAPQPPPPAETGKGAKEPSPRTNPVLNRPPEKRQRVPSAYNRFIKDEIQRIKAGNPDISHREAFSAAAKNWAHFPHIHFGLMPDHLGLKKTSLLPQRKEGLLKEGLLKDGLYAAATAAAANMGVAPY |
| PH02Gene11723.t1 | PeYABBY11 | MDLASPSEHLCYVRCTYCNTVLALQVGVPCKRLMDTVTVKCGHCNNLSFLRPRPQMVQPLSPTDHPMGPFQGPCNDCRRNQPLPLASPTSSEASPRAPFVVKPPEKKHRLPSAYNRFMREEIQRIKAAKPDIPHREAFSMAAKNWAKCDPRCSSTVSTSNSNEESRVMPGPQESDNEQVVESFDIFKQMERSG |
| PH02Gene30480.t1 | PeYABBY10 | MSAQIAPAPAEHVCYVHCNFCNTILAVSVPSNSMLNIVTVRCGHCTSLLSVNLRGLMQSLPVQDHFQENFKIHNISFSENYSEYGSSSKYRMPMMFSTLQSDPENMLHVRPPEKRQRVPSAYNRFIKEEIRRIKANNPDISHREAFSTAAKNWAHFPNIHFGLGSHESSKKLDEAIAAPGPQKVQGLY |
| PH02Gene29867.t1 | PeYABBY13 | MDLVSPSEHLCYVRCTYCNTVLAVGVPCKRLMDTVTVKCGHCNNLSFLSPRPPMVQPLSPTDHPMGPFQGPCNDCRRNQPLPLASPTSSDASPRAPFVVKPPEKKHRLPSAYNRFMREEIQRIKAAKPDIPHREAFSMAAKNWAKCDPRCSSTVSTSNSNQESRVVPGPQERSNEQVIESFDIFKQMERSG |
| PH02Gene30849.t1 | PeYABBY12 | MSAQIAPAPAEHVCYVHCNFCNTILAVSVPSNSMLNIVTVRCGHCNSLLSVNLRGLIQSLPVQDHFQENFKIHNISFGENYSEYGSSSKYRMPMMFSTESDPERMLHVRPPDQKRQRVPSAYNRFIKEEIRRIKANNPDISHREAFSTAAKNWAHFPNIHSGLGSHESSKKLDEAIDAPGPQKVQGLY |
| PH02Gene09731.t1 | PeYABBY14 | MMSSEAPETFSLDHLAQQQPPAEQDQLCYVHCNFCDTILAVGVPCSSMFKTVTVRCGHCANLLSVNLRGLLPAAAANQLPFGQALLSPTSPHGLLDELPFQAPPCLLTAEQTSANVSSNNSCANNTTATSMPPAKAAQREPQLPKSVPSATRPAEKRQRVPSAYNRFIKDEIQRIKASNPDITHREAFSAAAKNWAHFPHIHFGLMPDQGLKKTSIQSQDGTGECMLFKDGLYAAAAAAAATAASSMGVAPF |
| PH02Gene02055.t1 | PeYABBY16 | MSSAAPETFSLDHLAQQQQPPAEQEQLCYVHCNFCDTILAVGVPCSSLFKTVTVRCGHCTNLLSVNLRGLLLPAAAAAANQLPFGQALLSPTSPHGIIDEAPFQAPPSLLTEQASANVSSITSSNSSCANNTPATSMPPAKAAQREPQLPKSVPSATRPAEKRQRVPSAYNRFIKDEIQRIKASNPDITHREAFSAAAKNWAHFPHIHFGLMPDQGLKKTSIQSQDGAGECMLFKDGLYAAAAAAAASTASSMGITPF |
| PH02Gene16897.t1 | PeYABBY15 | MSSSSSSSAAFPLDHLAPSPTEQLCYVHCNCCDTILAVGVPCSSLFKTVTVRCGHCANLLSVNLRGLLLPPAEPPANQLHFGHSLLSPTSPHGLLDEVAFQTPSLLMDQASANLSSITGRSNNSCASNVPAMPMPMPGAKPAQQEPELPKSAPSANRPPEKRQRVPSAYNRFIKDEIQRIKAGNPDITHREAFSAAAKNWAHFPHIHFGLTPDQGLKKTFMPQDGAEDMLLKDGLYAAAAAAAAAEAANMGLTPF |

**Table S3 Gene expression profiles of *PeYABBY* genes**

| ID | ID Rename | Leaf | Panicle | Rhizome | Root |
| --- | --- | --- | --- | --- | --- |
| PH02Gene42615.t2 | PeYABBY01 | 8.48 | 1.12 | 4.375 | 0 |
| PH02Gene08310.t1 | PeYABBY03 | 56.15 | 20.08 | 14.61 | 0.17 |
| PH02Gene41238.t1 | PeYABBY02 | 0 | 0 | 0 | 0 |
| PH02Gene30423.t1 | PeYABBY04 | 13.7 | 1.43 | 2.61 | 0.06 |
| PH02Gene17872.t1 | PeYABBY05 | 0 | 0.76 | 0 | 0 |
| PH02Gene42853.t1 | PeYABBY06 | 47.625 | 64.285 | 13.205 | 0.72 |
| PH02Gene18063.t1 | PeYABBY07 | 11.83 | 1.6 | 3.015 | 0 |
| PH02Gene21516.t2 | PeYABBY08 | 81.235 | 111.445 | 17.76 | 0.295 |
| PH02Gene35716.t2 | PeYABBY09 | 3.645 | 9.755 | 4.34 | 2.36 |
| PH02Gene11723.t1 | PeYABBY11 | 4.645 | 16.215 | 0.63 | 0 |
| PH02Gene30480.t1 | PeYABBY10 | 59.62 | 54.975 | 40.285 | 0.31 |
| PH02Gene29867.t1 | PeYABBY13 | 12.455 | 26.11 | 2.005 | 0 |
| PH02Gene30849.t1 | PeYABBY12 | 35.79 | 34.025 | 31.335 | 0 |
| PH02Gene09731.t1 | PeYABBY14 | 24.225 | 1.455 | 6.395 | 0 |
| PH02Gene02055.t1 | PeYABBY16 | 16.885 | 1.425 | 4.06 | 0 |
| PH02Gene16897.t1 | PeYABBY15 | 6.815 | 3.94 | 1.75 | 0 |

**Notes.**

TPM for gene family expression profile calculation, the value of this expression is the average of duplicate data.

**S4 Table Primer for qRT-PCR.**

| Primer Name | Sequences (5' to 3') |
| --- | --- |
| PeYABBY01F | TATGGGATACCTGAACCCTAAC |
| PeYABBY01R | GTTGCTTCTTGCTCTGAACTCT |
| PeYABBY02F | TCTGCACCATCTTGCTGGTGAGC |
| PeYABBY02R | ACAAGGAAAGGAAGCCGGGGAT |
| PeYABBY03F | TGAGATTAGCCACAGGGAGG |
| PeYABBY03R | GAAATGGAGCCGAGGAAGAT |
| PeYABBY04F | CCGCTTCATCAAGGACGAAATCCA |
| PeYABBY04R | GAGACCGTGGTGATCCGGCATG |
| PeYABBY05F | CTCTTCTCCGTCAGCCTGTCTT |
| PeYABBY05R | CCCCACGTCGTCTCCACTGCTC |
| PeYABBY06F | GCAGCAAGTGTCTCCAATACG |
| PeYABBY06R | ATGCCTCCCTGTGGCTAATC |
| PeYABBY07F | TCAGCAAGGTCCATAGCGTC |
| PeYABBY07R | CGTTTTGTGCCGAGTACATCAT |
| PeYABBY08F | GGTTGCCTATGATGTACTCGG |
| PeYABBY08R | GGAACTCGTTGCCTCTTGTC |
| PeYABBY09F | CTGATCGCCGGTGTAGGTAA |
| PeYABBY09R | CTTGATGCGTTGGATTTCGT |
| PeYABBY10F | ACGGAGGATAAAAGCAAACAACCC |
| PeYABBY10R | GGAGCCCAGCCCAAAATGAA |
| PeYABBY11F | TCAGGGACCCTGCAATGACT |
| PeYABBY11R | TGGGAGGCGGTGTTTCTTCT |
| PeYABBY12F | GAATGCCGATGATGTTCTCA |
| PeYABBY12R | TCTGTGGCTTATGTCAGGGTT |
| PeYABBY13F | CCCATCTGCTTACAATCGCTTCA |
| PeYABBY13R | TCGCACTTCGCCCAGTTCTT |
| PeYABBY14F | AGAGTTCCCTCGGCTTACAA |
| PeYABBY14R | TCAGACCGAAATGAATGTGC |
| PeYABBY15F | GAGTCCCATCGGCGTACAAT |
| PeYABBY15R | CATCCTGAGGCATGAAGGTC |
| PeYABBY16F | AGAGTTCCCTCGGCATACAA |
| PeYABBY16R | AAAGCCTCCCTGTGGGTGAT |
| NTB-F | TCTTGTTTGACACCGAAGAGGAG |
| NTB-R | AATAGCTGTCCCTGGAGGAGTTT |

**Table S5 The gene name of the protein interaction network of Moso bamboo and *[Arabidopsis](C:/Users/Administrator/AppData/Local/youdao/dict/Application/8.9.3.0/resultui/html/index.html#/javascript:;)***

| ID | ID Rename | Subfamily |
| --- | --- | --- |
| PH02Gene42615.t2 | PeYABBY01 | AFO(FIL) |
| PH02Gene41238.t1 | PeYABBY02 | YAB5 |
| PH02Gene08310.t1 | PeYABBY03 | YAB2 |
| PH02Gene30423.t1 | PeYABBY04 | AFO(FIL) |
| PH02Gene17872.t1 | PeYABBY05 | NA |
| PH02Gene42853.t1 | PeYABBY06 | YAB2 |
| PH02Gene18063.t1 | PeYABBY07 | YAB5 |
| PH02Gene21516.t2 | PeYABBY08 | YAB2 |
| PH02Gene35716.t2 | PeYABBY09 | AFO(FIL) |
| PH02Gene30480.t1 | PeYABBY10 | YAB5 |
| PH02Gene11723.t1 | PeYABBY11 | CRC |
| PH02Gene30849.t1 | PeYABBY12 | YAB5 |
| PH02Gene29867.t1 | PeYABBY13 | CRC |
| PH02Gene09731.t1 | PeYABBY14 | AFO(FIL) |
| PH02Gene16897.t1 | PeYABBY15 | AFO(FIL) |
| PH02Gene02055.t1 | PeYABBY16 | AFO(FIL) |

| Name | *Arabidopsis* ID | *P.edulis* ID |
| --- | --- | --- |
| AS1 | AT2G37630 | PH02Gene11804.t1 |
| AS2 | AT1G65620 | PH02Gene43858.t1 |
| ETT | AT2G33860 | PH02Gene22930.t1 |
| KAN2 | AT1G32240 | PH02Gene39580.t2 |
| KAN | AT5G16560 | PH02Gene47465.t1 |

**S6 Table GO annotation analysis of *PeYABBY* genes**

| Description | Number | GO ID | Term Type | Padjust |
| --- | --- | --- | --- | --- |
| abaxial cell fate specification | 8 | GO:0010158 | BP | 6.36761E-05 |
| cellular developmental process | 12 | GO:0048869 | BP | 0.000120754 |
| developmental process involved in reproduction | 7 | GO:0003006 | BP | 0.000120754 |
| specification of animal organ position | 2 | GO:0010159 | BP | 0.000120754 |
| style development | 2 | GO:0048479 | BP | 0.000120754 |
| nucleus | 11 | GO:0005634 | CC | 0.000253799 |
| multicellular organism development | 9 | GO:0007275 | BP | 0.000276584 |
| cell fate specification | 8 | GO:0001708 | BP | 0.000327324 |
| regionalization | 4 | GO:0003002 | BP | 0.000327324 |
| reproductive process | 7 | GO:0022414 | BP | 0.000464223 |
| anatomical structure development | 10 | GO:0048856 | BP | 0.000464223 |
| cell differentiation | 7 | GO:0030154 | BP | 0.000464223 |
| inflorescence meristem growth | 2 | GO:0010450 | BP | 0.000464223 |
| multicellular organismal process | 12 | GO:0032501 | BP | 0.000465128 |
| pattern specification process | 5 | GO:0007389 | BP | 0.000465128 |
| developmental process | 14 | GO:0032502 | BP | 0.000465128 |
| nectary development | 2 | GO:0010254 | BP | 0.000745256 |
| specification of floral organ number | 2 | GO:0048833 | BP | 0.002742053 |
| metal ion binding | 11 | GO:0046872 | MF | 0.002742053 |
| cation binding | 11 | GO:0043169 | MF | 0.002742053 |
| specification of plant organ number | 2 | GO:0048832 | BP | 0.003442642 |
| carpel development | 2 | GO:0048440 | BP | 0.004973782 |
| floral meristem determinacy | 2 | GO:0010582 | BP | 0.006919741 |
| specification of plant organ identity | 2 | GO:0090701 | BP | 0.008119403 |
| specification of floral organ identity | 2 | GO:0010093 | BP | 0.008119403 |
| meristem growth | 2 | GO:0035266 | BP | 0.00871814 |
| meristem determinacy | 2 | GO:0010022 | BP | 0.00871814 |
| meristem structural organization | 2 | GO:0009933 | BP | 0.013047513 |
| fruit development | 2 | GO:0010154 | BP | 0.013331252 |
| regulation of developmental process | 4 | GO:0050793 | BP | 0.017263867 |
| anatomical structure arrangement | 2 | GO:0048532 | BP | 0.018807856 |
| floral organ development | 2 | GO:0048437 | BP | 0.03886968 |
| plant organ development | 3 | GO:0099402 | BP | 0.13402169 |
| intracellular membrane-bounded organelle | 11 | GO:0043231 | CC | 0.204259365 |
| leaf development | 2 | GO:0048366 | BP | 0.275850313 |
| ion binding | 11 | GO:0043167 | MF | 0.298768286 |
| regulation of flower development | 2 | GO:0009909 | BP | 0.348842167 |
| phyllome development | 2 | GO:0048827 | BP | 0.398731673 |
| regulation of shoot system development | 2 | GO:0048831 | BP | 0.474047206 |
| reproductive structure development | 2 | GO:0048608 | BP | 0.760735763 |
| regulation of reproductive process | 2 | GO:2000241 | BP | 0.765609546 |
| membrane-bounded organelle | 11 | GO:0043227 | CC | 0.941974112 |
| regulation of post-embryonic development | 2 | GO:0048580 | BP | 1 |
| developmental growth | 2 | GO:0048589 | BP | 1 |
| polarity specification of adaxial/abaxial axis | 1 | GO:0009944 | BP | 1 |
| specification of axis polarity | 1 | GO:0065001 | BP | 1 |
| regulation of multicellular organismal development | 2 | GO:2000026 | BP | 1 |
| growth | 2 | GO:0040007 | BP | 1 |
| regulation of multicellular organismal process | 2 | GO:0051239 | BP | 1 |
| plant ovule development | 1 | GO:0048481 | BP | 1 |
| cellular process | 12 | GO:0009987 | BP | 1 |
| biological_process | 14 | GO:0008150 | BP | 1 |
| intracellular organelle | 11 | GO:0043229 | CC | 1 |
| organelle | 11 | GO:0043226 | CC | 1 |
| DNA binding | 4 | GO:0003677 | MF | 1 |

1. Description: the description of the GO function. (2)Number: the number of genes enriched to the GO term; (3) GO ID: the number corresponding to the GO Term; (4) Term Type: the three major classifications of GO (i.e. BP, CC, MF); (5) Padjust: Padjust represents whether the enriched result is statistically significant or not, the smaller the Padjust, the more statistically significant it is.
